# The coordinated action of VCP/p97 and GCN2 regulates cancer cell metabolism and proteostasis during nutrient limitation

**DOI:** 10.1038/s41388-018-0651-z

**Published:** 2019-01-09

**Authors:** Katarzyna Parzych, Paula Saavedra-García, Gabriel N. Valbuena, Hibah A. Al-Sadah, Mark E. Robinson, Lucy Penfold, Desislava M. Kuzeva, Angie Ruiz-Tellez, Sandra Loaiza, Viktoria Holzmann, Valentina Caputo, David C. Johnson, Martin F. Kaiser, Anastasios Karadimitris, Eric W-F Lam, Eric Chevet, Niklas Feldhahn, Hector C. Keun, Holger W. Auner

**Affiliations:** 1 Cancer Cell Protein Metabolism Group, Centre for Haematology, Department of Medicine, Imperial College London, London, UK; 2 Division of Cancer, Department of Surgery and Cancer, Imperial College London, Hammersmith Hospital, London, UK; 3 Centre for Haematology, Department of Medicine, Imperial College London, London, UK; 4 Division of Molecular Pathfology, Institute of Cancer Research, Sutton, UK; 5 INSERM U1242, Chemistry, Oncogenesis, Stress, Signaling, Université de Rennes 1, Rennes, France; 6 Centre de Lutte Contre le Cancer Eugène Marquis Rennes, Rennes, France

**Keywords:** Cancer metabolism, Ubiquitylation, Nutrient signalling

## Abstract

VCP/p97 regulates numerous cellular functions by mediating protein degradation through its segregase activity. Its key role in governing protein homoeostasis has made VCP/p97 an appealing anticancer drug target. Here, we provide evidence that VCP/p97 acts as a regulator of cellular metabolism. We found that VCP/p97 was tied to multiple metabolic processes on the gene expression level in a diverse range of cancer cell lines and in patient-derived multiple myeloma cells. Cellular VCP/p97 dependency to maintain proteostasis was increased under conditions of glucose and glutamine limitation in a range of cancer cell lines from different tissues. Moreover, glutamine depletion led to increased VCP/p97 expression, whereas VCP/p97 inhibition perturbed metabolic processes and intracellular amino acid turnover. GCN2, an amino acid-sensing kinase, attenuated stress signalling and cell death triggered by VCP/p97 inhibition and nutrient shortages and modulated ERK activation, autophagy, and glycolytic metabolite turnover. Together, our data point to an interconnected role of VCP/p97 and GCN2 in maintaining cancer cell metabolic and protein homoeostasis.

## Introduction

Cancer cells have a heightened dependency on the mechanisms of protein degradation to maintain protein homoeostasis (proteostasis) [1]. Therefore, drugs that disrupt protein breakdown and trigger proteotoxic stress have considerable potential for anticancer therapy [2, 3]. This proposition is supported by the clinical success of inhibitors of the proteasome, the main effector of intracellular protein degradation, in multiple myeloma (MM) [4]. VCP/p97 is an AAA+ (ATPase associated with diverse cellular activities) ATPase that is conserved across diverse species, is essential for life in budding yeast and mice, and is abundantly expressed [5–8]. VCP/p97 exerts a segregase activity that uses energy derived from ATP hydrolysis to remodel client proteins, and engages in multiple cellu lar processes that include chromosome segregation, DNA repair, endoplasmic reticulum (ER) and Golgi formation, and activation of NF-κB [9–16]. Its role is best understood in the context of ER-associated degradation (ERAD), where VCP/p97 mediates the delivery of ubiquitinated misfolded ER proteins to the proteasome [17–19]. However, VCP/p97 has also been linked to proteasome-independent handling of protein aggregates and autophagy [20–23]. Moreover, VCP/p97 has been implicated in proteasome recovery after proteasome inhibition [24–26]. This broad involvement of VCP/p97 in regulating protein homoeostasis suggests that VCP inhibitors could overcome therapeutic limitations of proteasome inhibitors [1, 27]. Indeed, VCP/p97 inhibitors trigger fatal ER stress in cancer cells in vitro and in vivo [22, 28–30]. Increased expression levels of VCP/p97 have been observed in multiple cancers and may explain the preferential toxicity of VCP/p97 inhibition in cancerous compared to non-malignant cell lines [22, 31–33]. Recently, anticancer effects of the alcohol-abuse drug disulfiram have been linked to VCP/p97 and its adaptor NPL4 [34]. Thus, VCP/p97 and its adaptors are promising therapeutic targets in oncology [29, 35, 36]. However, the functional links between VCP/p97 and cellular metabolism, and the effects of nutrient availability on cancer cells targeted by VCP/p97 inhibition, remain to be established. Additional therapeutic relevance of such interactions is highlighted by the observation that VCP/p97 promotes degradation of glutamine synthetase (GS) downstream of CRBN, the target of so-called immunomodulatory drugs [37].

Cancer cells adapt their metabolic networks to reconcile the need for increased anabolic biomass synthesis and catabolic energy production with suboptimal nutrient supplies [38–40]. The reprogramming of cellular metabolism can confer survival advantages to tumour cells but also offers therapeutic opportunities [41]. Glutamine and glucose are two principal nutrients that support cell growth and survival, and the high demand of cancer cells for both molecules has been known for many decades [42–44]. Moreover, limited availability of glutamine and glucose in tumours may have a detrimental effect on therapeutic responses [45–52]. The amino acid-sensing kinase GCN2 (EIF2AK4) provides a direct link between nutrient availability and protein homoeostasis [53]. Upon activation by uncharged tRNAs or ribosome stalling, GCN2 phosphorylates its only known target, eIF2α, resulting in attenuation of overall protein synthesis [54–56]. GCN2 also regulates amino acid homoeostasis through sestrin2-mediated repression of mTORC1 and induction of autophagy [57–60]. GCN2 supports cancer cell survival under conditions of amino acid or glucose deprivation but may also promote cell death upon prolonged nutrient depletion [61–63].

Here, we present evidence that VCP/p97 acts as a central regulator of cellular metabolism. Furthermore, we uncover a role for GCN2 to promote cell viability during nutrient deprivation and VCP/p97 inhibitor-induced proteotoxic stress. Together, our data point to a pivotal and interconnected role of VCP/p97 and GCN2 in maintaining cancer cell metabolic and protein homoeostasis.

## Results

### Cancer cell dependency on VCP/p97 is linked to metabolic processes and nutrient availability

**Fig. 1:**
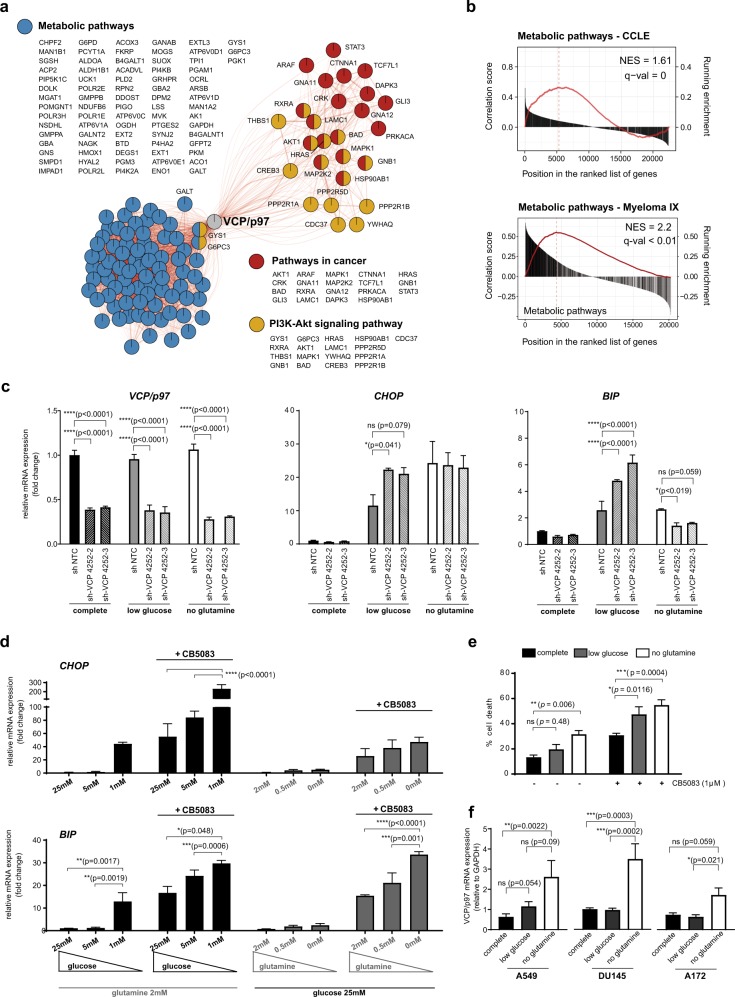
VCP/p97-governed proteostasis is tied to metabolic pathways and nutrient availability. **a** Network graph of leading-edge enriched genes from the top three most highly enriched KEGG pathways. Edge weight and size indicate strength of correlation between genes/nodes. Nodes are coloured by the pathways each gene contributes to. **b** GSEA plots of the KEGG term Metabolic Pathways, with genes ranked by correlation with VCP/p97 expression, in the Cancer Cell Line Encyclopedia (CCLE) and in patient-derived tumour cells in the Myeloma IX study. The correlation value of individual genes is indicated by black bars (left axis) and running enrichment score is highlighted by red line (right axis). NES normalised enrichment score. **c** Relative mRNA levels of the indicated genes in A549 cells stably expressing non-targeting control (NTC) or one of two IPTG-inducible VCP/p97 shRNAs after 48 h of IPTG treatment in complete medium (25 mM glucose, 2 mM glutamine), followed by 24 h maintenance in complete, low glucose (1 mM glucose, 2 mM glutamine) or no glutamine (25 mM glucose, 0 mM glutamine) medium supplemented with 500 μM IPTG. Data shown are mean and SEM, *n* = 3 (two-way ANOVA with Tukey’s multiple comparisons test). **d** Relative mRNA levels of the indicated genes in A549 cells grown under different conditions of nutrient availability and treated with CB5083 (1 μM) as indicated for 16 h. Data shown are mean and SEM, *n* = 3 (two-way ANOVA with Tukey’s multiple comparisons test). **e** Proportion of non-viable A549 cells as determined by Annexin V and 7-AAD staining after treatment with CB5083 under the indicated conditions of nutrient availability for 48 h. Data shown are mean and SEM, *n* = 3 (two-way ANOVA with Sidak’s multiple comparisons test). **f** VCP/p97 mRNA expression in the indicated cell lines grown in complete, low glucose or no glutamine medium for 16 h (A549), 24 h (DU145), or 48 h (A172). Data shown are mean and SEM, *n* = 3 (Mann–Whitney test)

To investigate whether VCP/p97 is linked to cancer cell metabolism, we first interrogated the Cancer Cell Line Encyclopedia (CCLE) [64]. We found that VCP/p97 mRNA expression across 917 CCLE cancer cell lines significantly correlated with 162 Kyoto Encyclopedia of Genes and Genomes (KEGG) terms. As expected, these included several processes linked to protein folding, sorting, and degradation such as ‘ubiquitin-mediated proteolysis’, ‘protein processing in endoplasmic reticulum’, and ‘proteasome’. When ranked by significance levels, the top three KEGG terms linked to VCP/p97 expression were ‘metabolic pathways’, ‘pathways in cancer’, and ‘PI3K-Akt signalling pathway’ (Fig. 1a and Supplementary Table 2). VCP/p97 expression was also significantly correlated with 22 cancer-specific pathways linked to both solid and haematopoietic malignancies, and with 14 metabolic pathways including ‘glycolysis/gluconeogenesis’, ‘carbon metabolism’, and ‘biosynthesis of amino acids’. Moreover, VCP/p97 expression correlated with several processes that are linked to cellular metabolism and are frequently deregulated in cancer, such as ‘mTOR signalling pathway’ and ‘MAPK signalling pathway’ (Supplementary Table 2). Next, we tested if VCP/p97 expression is linked to metabolic pathways in purified bone marrow tumour cells of 261 patients with MM [65]. Out of 76 KEGG terms that correlated significantly with VCP/p97 expression, which contained ‘ubiquitin-mediated proteolysis’, ‘protein processing in endoplasmic reticulum’, and ‘proteasome’, 32 terms were metabolic pathways that included central processes such as ‘citrate cycle (TCA cycle)’ and ‘carbon metabolism’ (Supplementary Table 3). As with the CCLE data, the top co-regulated KEGG pathway was ‘metabolic pathways’ (Fig. 1b). Taken together, these data demonstrate that VCP/p97 is, at the level of gene expression, tied to multiple metabolic pathways, including several that are directly linked to the processing of nutrients, in a diverse range of cancers.

We then set out to investigate the link between VCP/p97-guarded protein homoeostasis and nutrient availability in cultured cells. First, we knocked down VCP/p97 in A549 lung adenocarcinoma cells using stable inducible small hairpin RNAs (shRNAs) and quantified mRNA levels of BIP and CHOP as markers of the unfolded protein response (UPR) and integrated stress response (ISR), respectively, after glutamine or glucose depletion [66–68]. We observed that shRNA-mediated VCP/p97 mRNA reduction by approximately 60% did not significantly alter CHOP or BIP transcript levels in cells maintained in complete medium (Fig. 1c), indicating that the remaining VCP/p97 activity was sufficient to maintain proteostasis under conditions of optimal nutrient supply. Depletion of glucose or glutamine in control (non-targeting shRNA) cells resulted in higher levels of both CHOP and BIP mRNAs, indicating that both glucose and glutamine limitation perturbed proteostasis. Compared to control cells, CHOP and BIP mRNAs were significantly more upregulated in VCP/p97 knockdown cells exposed to low glucose conditions, but not in VCP/p97 knockdown cells depleted of glutamine (Fig. 1c). Considering that an inducible knockdown reduces mRNA and protein levels gradually and incompletely, potentially allowing cells to adapt to a reduction of VCP/p97 activity, we turned to CB5083, a selective inhibitor of the VCP/p97 ATPase that has been undergoing early preclinical and clinical testing in solid and haematopoietic malignancies (NCT02243917, NCT02223598) [29, 35]. In parental A549 cells, glucose restriction induced BIP (13-fold) and CHOP mRNAs (44-fold) considerably more than glutamine depletion (2.5-fold and 5-fold, respectively). We also observed CB5083-induced upregulation of BIP (16-fold) and CHOP (55-fold) mRNAs in cells grown in complete medium. This was increased further by glucose and glutamine depletion, although the additional CHOP induction in glutamine-depleted cells was not statistically significant (Fig. 1d). Consistent with the increased expression of CHOP and BIP mRNAs, nutrient depletion also increased the levels of CB5083-induced cell death in A549 cells (Fig. 1e). Glutamine depletion and, to some extent, glucose limitation, also resulted in higher levels of CB5083-induced cell death in PC3 prostate cancer, MCF-7 breast cancer, and Saos-2 osteosarcoma cell (Supplementary Figure 1). Moreover, glucose and glutamine depletion significantly increased CB5083-induced caspase activation in A549 cells (Supplementary Figure 2). This effect of nutrient depletion on caspase activation was also observed in other cell types, including DU145 and PC3 prostate cancer and MCF-7 breast cancer cells, albeit at different levels, and with glutamine depletion generally having a more pronounced effect than glucose limitation (Supplementary Figure 2). Caspase inhibition with the pan-caspase inhibitor z-VAD-fmk reduced cell death caused by CB5083 treatment and nutrient depletion (Supplementary Figure 3). Thus, depletion of glucose and glutamine increases proteotoxic stress that is at least partly ER-related and triggers partly caspase-dependent apoptotic death, upon genetic or pharmacological VCP/p97 depletion.

We then tested whether suboptimal nutrient availability would alter VCP/p97 expression and observed that glutamine depletion upregulated VCP/p97 mRNA in A549, DU145, and A172 (glioblastoma) cells (Fig. 1f). However, we did not detect an unequivocal increase in VCP/p97 protein levels by immunoblotting (Supplementary Figure 4), possibly because of high baseline expression levels and limited sensitivity of the approach. Glucose restriction did not appear to trigger VCP/p97 mRNA upregulation (Fig. 1f). Taken together, the results indicate increased cellular VCP/p97 dependency to maintain proteostasis when nutrient supplies are suboptimal. The data also suggest that glutamine depletion triggers compensatory mechanisms that appear to be more effective in attenuating stress than those induced by glucose depletion. Finally, we also determined that VCP/p97 mRNA or protein expression did not correlate with cellular sensitivity to CB5083 (Supplementary Figure 5), a finding compatible with our previous observations in a panel of cancer cell lines largely composed of MM cells and treated with the VCP/p97 inhibitors DBeQ and NMS-873 [30]. However, the degree to which BIP and CHOP mRNAs are upregulated in A549 cells in response to CB5083 is concentration dependent, as is the degree of cell death in response to CB5083 in a range of cancer cells (Supplementary Figure 6). Thus, VCP/p97 inhibition kills cancer cells in a dose-responsive manner.

### VCP/p97 inhibition perturbs key metabolic pathways

**Fig. 2:**
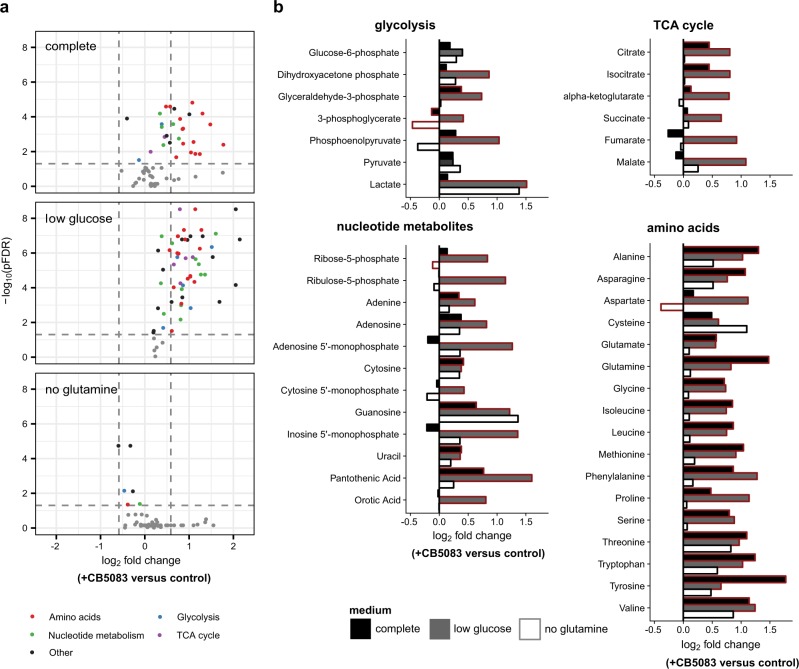
Metabolite responses to VCP/p97 inhibition. A549 cells were grown in complete (25 mM glucose, 2 mM glutamine), low glucose (1 mM glucose, 2 mM glutamine), or no glutamine (25 mM glucose, 0 mM glutamine) medium for 16 h followed by intracellular metabolite extraction and gas chromatography-mass spectrometry. Data show the effects of CB5083 treatment (1 μM) for 16 h. **a** Volcano plots indicating metabolites with significant changes (coloured). The horizontal dashed line represents a multiple testing-adjusted *p*-value of 0.05, and the vertical dashed lines represent log_2_ fold changes of 0.67 and 1.5, respectively. **b** Bar graphs show the effect of treatment with CB5083 on individual metabolites (fold change of treatment to control for each of the three media compositions). Comparisons with significant effects (two-way ANOVA, *n* = 4 per experimental condition) are outlined in red

To further investigate how VCP/p97 regulates cellular metabolism, we used gas chromatography–mass spectrometry (GC–MS) to quantify intracellular metabolites in cells treated with CB5083 under different conditions of nutrient availability. As expected, glucose depletion alone resulted in significant decreases in glycolytic metabolites, and in decreased levels of some nucleotide metabolites and amino acids (Supplementary Figure 7). Glutamine depletion also affected glycolytic and nucleotide metabolites, although to a lesser extent than glucose limitation, and was linked to significant decreases in levels of TCA cycle intermediates and of some amino acids, including glutamine and glutamate. In contrast, inhibition of VCP/p97 in A549 cells grown in complete medium was predominantly linked to significant increases in the levels of most amino acids, with tyrosine and glutamine being the most affected (Fig. 2a, b). CB5083 treatment also triggered increases of TCA cycle, glycolytic, and nucleotide metabolites. Somewhat intriguingly, when considering the increased toxicity of VCP/p97 inhibition in low glucose conditions, VCP/p97 inhibition largely countered the effects of glucose limitation on metabolites. Compared to low glucose conditions alone, CB5083 treatment significantly increased the intracellular levels of most nucleotide metabolites, glycolytic intermediates, TCA cycle components, and amino acids (Fig. 2a, b). In sharp contrast, the effects of VCP/p97 inhibition in glutamine-depleted cells were small or insignificant. These results provide further evidence for an important role of VCP/p97 in regulating intracellular metabolism and support the notion that mechanisms to compensate for the metabolic effects of pharmacological VCP/p97 inhibition are activated under glutamine-limiting conditions.

### GCN2 attenuates stress signalling triggered by VCP/p97 inhibition and glutamine deprivation

**Fig. 3:**
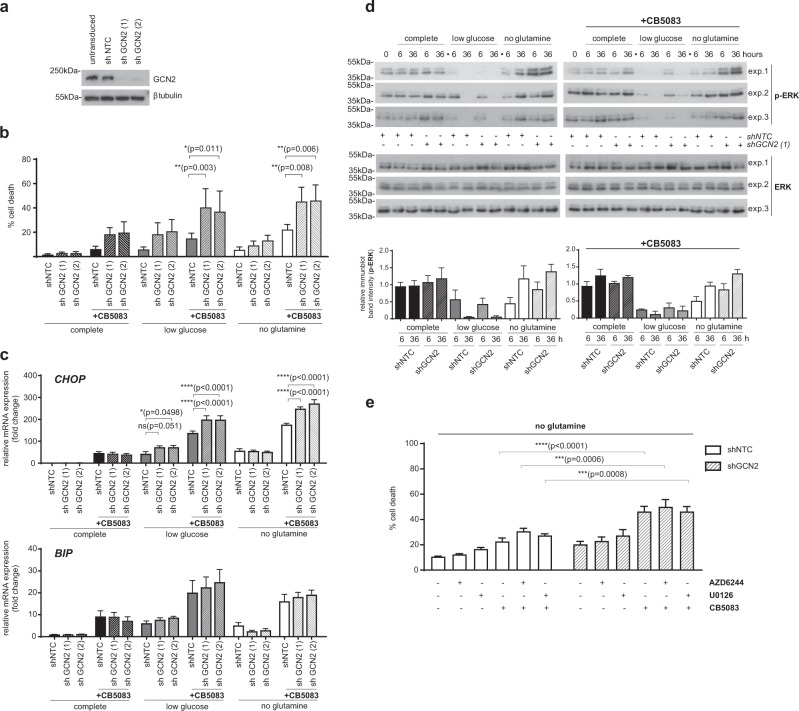
GCN2 modulates proteotoxic stress and ERK signalling in response to VCP/p97 inhibition. **a** Immunoblot for GCN2 and β-tubulin on extracts from untransduced A549 cells and A549 cells stably expressing targeting (shGCN2) or non-targeting (shNTC) shRNA**. b-e** A549 cells stably expressing non-targeting control (shNTC) or one of two shRNAs targeting GCN2 (shGCN2) were grown in complete (25 mM glucose, 2 mM l-glutamine), low glucose (1 mM glucose, 2 mM glutamine), or no glutamine (25 mM glucose, 0 mM glutamine) medium. CB5083 was used at 1 μM. **b** Proportion of non-viable A549 cells as determined by Annexin V-FITC and 7-AAD staining after 48 h of experimental treatment. Data shown are mean and SEM, *n* = 3 (two-way ANOVA with Tukey’s multiple comparisons test). **c** mRNA levels relative to shNTC expressing cells grown in complete medium after 16 h of experimental treatment. Data shown are mean and SEM, *n* = 3 (two-way ANOVA with Tukey’s multiple comparisons test). **d** Immunoblot analyses on whole-cell extracts from A549 cells (three independent experiments) stably expressing shNTC or shGCN2(1). Bar graphs show relative phosphorylated ERK1/2 immunoblot band intensities (mean and SEM) compared to control cells. **e** Proportion of non-viable A549 cells as determined by Annexin V and 7-AAD staining after 24 h of experimental treatment with AZD6244, U0126, and CB5083 for 24 h. Mean and SEM of *n* = 3 experiments is shown (two-way ANOVA and Tukey’s multiple comparisons for effects of AZD6244 and U0126 compared to vehicle-treated or CB5083-treated cells; two-way ANOVA and Bonferroni’s multiple comparisons test for effects of shGCN2 compared to shNTC under the same experimental conditions (grey asterisks); exact *p* values for all comparisons are shown in Supplementary Figure 10)

We have previously shown that VCP/p97 inhibition triggers time- and concentration-dependent phosphorylation of GCN2 on Thr889, suggesting its activation [30]. Observations that GCN2 may promote tumour growth or induce apoptosis under conditions of metabolic stress and nutrient deprivation led us to investigate the role of GCN2 in cells challenged by VCP/p97 inhibition [61, 63, 68–70]. We first determined the effect of GCN2 depletion by stable shRNA-mediated knockdown (Fig. 3a) on cell viability. VCP/p97 inhibition, nutrient depletion, or both together consistently triggered higher levels of death of GCN2-depleted cells than of control cells. However, the differences were only statistically significant when cells were stressed by both CB5083 treatment and nutrient depletion (Fig. 3b). Under these conditions, cell death rates were approximately twice as high in shGCN2 cells compared to control cells. GCN2-depleted cells also expressed higher levels of CHOP mRNA than control cells in low glucose medium, or when they were exposed to CB5083 under glucose- or glutamine-depleted conditions (Fig. 3c). BIP mRNA levels showed a similar trend as CHOP levels but the differences were small and not statistically significant. These observations suggest that, under the stress conditions tested, GCN2 promotes survival and attenuates stress signalling linked to CHOP expression but may not be directly linked to protein folding mechanisms in the ER. We also found higher levels of CB5083-induced caspase activation in GCN2-depleted A549 cells in glutamine-limiting conditions (Supplementary Figure 8). The findings also suggest that the survival-promoting role of GCN2 is most relevant under complex stress conditions of combined VCP/p97 inhibition and nutrient depletion. In line with this notion, we found that GCN2 mRNA was upregulated by CB5083, and that this increase in mRNA levels was further accentuated by glutamine depletion, although the changes were numerically small and did not reach statistical significance (Supplementary Figure 9).

GCN2 has been reported to mediate cell death under conditions of prolonged glucose restriction, and this pro-apoptotic function was linked to activation of ERK2 upstream of GCN2 [63]. However, we observed that glucose depletion reduced ERK1/2 phosphorylation rapidly and persistently (Fig. 3d). Glutamine depletion caused a transient reduction in ERK phosphorylation, and treatment with CB5083 did not have a discernible effect on ERK phosphorylation, irrespective of nutrient availability. While GCN2 depletion had no distinct effect on ERK phosphorylation in cells grown in complete or low glucose medium, we observed a trend towards higher levels of ERK phosphorylation in GCN2-depleted cells that were deprived of glutamine, irrespective of whether they were, or were not, treated with CB5083. To determine how ERK regulates cell fate under these stress conditions, we quantified cell viability after ERK inhibition in GCN2 competent and depleted cells grown without glutamine. First, providing further evidence for a pro-survival role of GCN2 under stress conditions, we observed significantly higher levels of cell death in GCN2-depleted cells treated with CB5083 compared to control cells (Fig. 3e and Supplementary Figure 10a). ERK inhibition with two small molecule inhibitors was generally linked to a numeric increase in cell death, although the effects were not statistically significant (Fig. 3e and Supplementary Figure 10b). Thus, we did not find that a pro-apoptotic ERK signalling pathway that involves GCN2 was active in our cellular model system and under the stress conditions tested. In contrast, GCN2, and to a small extent ERK, promoted survival.

### Autophagy promotes cell survival under complex stress conditions

**Fig. 4:**
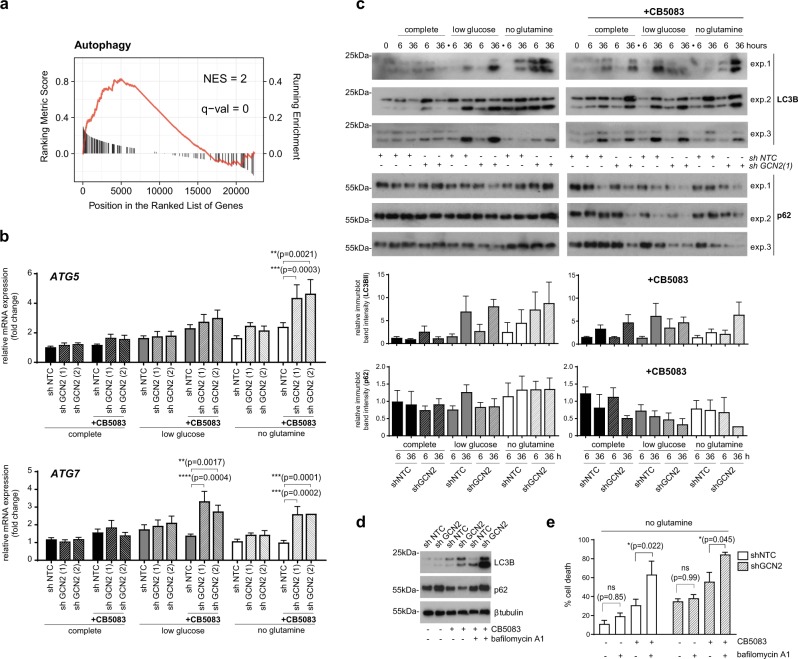
GCN2 attenuates autophagy in response to metabolic and proteotoxic stress. A549 cells stably expressing non-targeting control (shNTC) or one of two shRNAs targeting GCN2 (shGCN2) were grown in complete (25 mM glucose, 2 mM l-glutamine), low glucose (1 mM glucose, 2 mM glutamine), or no glutamine (25 mM glucose, 0 mM glutamine) medium. CB5083 was used at 1 μM. **a** GSEA plot of KEGG autophagy pathway with genes ranked by correlation with VCP/p97 expression. The correlation value of individual genes is indicated by black bars (left axis) and running enrichment score is highlighted by red line (right axis). NES normalised enrichment score. **b** mRNA levels relative to shNTC expressing cells grown in complete medium after 16 h of experimental treatment. Data shown are mean and SEM, *n* = 3 (two-way ANOVA with Tukey’s multiple comparisons test). **c** Immunoblot analyses on whole-cell extracts from A549 cells (three independent experiments) stably expressing shNTC or shGCN2(1). Bar graphs show relative LC3BII and p62 immunoblot band intensities, compared to control cells (mean and SEM). **d** Immunoblot analyses on whole-cell extracts from A549 cells treated with CB5083 (1 μM) for 24 h and bafilomycin A1 (100 nM) for 16 h. **e** Propoof non-viable A549 cells treated with CB5083 (1 μM) for 48 h and bafilomycin A1 (100 nM) for 40 h. Data shown are mean and SEM, *n* = 3 (two-way ANOVA and Tukey’s multiple comparisons test)

Macroautophagy, thereafter referred to as autophagy, is a highly conserved intracellular pathway whose primary function is to sustain cellular metabolism during nutrient starvation. Autophagy has been linked to VCP/p97 inhibition experimentally, albeit with conflicting findings as to whether VCP/p97 inhibition suppresses or induces autophagy [22, 29]. Autophagy has also been reported to be induced downstream of GCN2 [57–59, 71, 72]. We found that VCP/p97 expression is linked to autophagic processes across the CCLE (Fig. 4a). We also observed that ATG7 mRNA levels were significantly higher in GCN2-depleted cells grown in low glucose and no glutamine medium when treated with CB5083, while ATG5 mRNA was significantly upregulated only in GCN2-depleted cells grown in no glutamine medium and treated with CB5083 (Fig. 4b). We also observed that, as expected, both glutamine and glucose depletion induced autophagy when assessed by immunoblotting for LC3BII, at the 36 h time point (Fig. 4c). VCP/p97 inhibition with CB5083 also appeared to induce autophagy in cells maintained in complete and nutrient-depleted medium, based on higher LC3BII and lower p62 levels. While GCN2 depletion did not have a detectable impact on LC3B or p62 levels in most conditions tested, we observed a possible trend to higher LC3BII levels in shGCN2 compared to shNTC cells that were glutamine-depleted, irrespective of whether they were or were not treated with CB5083. GCN2 depletion was also linked to lower levels of p62 in CB5083-treated cells compared to control cells. Thus, while LC3BII levels were affected by nutrient depletion or VCP/p97 inhibition, p62 levels and key autophagy mRNAs were only affected when cells experienced a combined metabolic challenge of GCN2 depletion, VCP/p97 inhibition, and nutrient limitation, in particular glutamine depletion. We then set out to test if the effects of autophagy were cytoprotective by blocking autophagy for 16 h with bafilomycin A1 after an 8-h CB5083 pre-treatment of glutamine depleted cells. We confirmed the effects of CB5083 on LC3B and p62 levels at the single time point used in this experiment, and that these effects were enhanced in GCN2-depleted cells (Fig. 4d). Moreover, we found that treatment with bafilomycin A1 resulted in a stronger LC3BII immunoblot signal, which is consistent with an increased autophagic flux in the CB5083-treated cells. This effect of bafilomycin A1 was particularly pronounced in the GCN2-depleted cells, in keeping with higher levels of autophagy. When we determined cell viability, we found that bafilomycin A1 significantly increased cell death in CB5083-treated shNTC and shGCN2 cells, but did not have a significant effect on viability in cells not exposed to CB5083 (Fig. 4e). Taken together, the data indicate that autophagy is induced to limit the deleterious effects of combined glutamine depletion and VCP/p97 inhibition, and that this effect is particularly noticeable in GCN2-depleted cells. Thus, both autophagy and GCN2 play a role in maintaining cell survival under these stress conditions.

### GCN2 attenuates the depletion of glycolytic metabolites under low glucose conditions

**Fig. 5:**
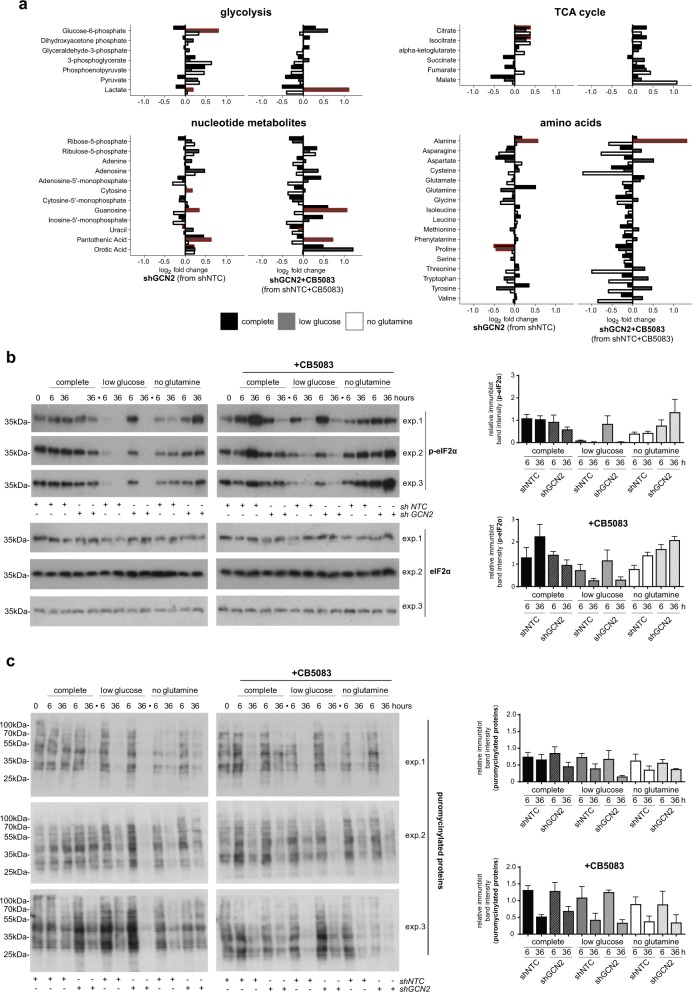
Effects of GCN2 on metabolites and protein synthesis. **a** Bar graphs show metabolite levels in shGCN2 A549 cells compared to shNTC cells in different media (fold change for each of the three media compositions (16 h), and metabolite levels in shGCN2(1) cells (fold change to shNTC cells) after treatment with CB5083 (1 μM, 16 h). Comparisons with significant effects are outlined in red (*n* = 4, two-way ANOVA). **b**, **c** Immunoblot analyses for the indicated proteins on whole-cell extracts from A549 cells (three independent experiments) stably expressing shNTC or shGCN2(1). Bar graphs show relative immunoblot band intensities, compared to control cells (mean and SEM)

Our findings that GCN2 promotes viability under conditions of proteotoxic and metabolic stress, in combination with the known roles of GCN2 in regulating protein synthesis via eIF2α and autophagy in response to amino acid shortages, prompted us to investigate the effect of GCN2 depletion on intracellular metabolites. Somewhat surprisingly, stable GCN2 knockdown was linked to very limited effects on metabolites in cells grown in complete or glutamine-depleted medium (Fig. 5a). In cells grown under low glucose conditions, GCN2 depletion was linked to significantly increased levels of some nucleotide metabolites, glucose-6-phosphate, and lactate. We also observed significantly increased alanine levels in GCN2-depleted cells, in keeping with higher lactate levels. Significant effects of GCN2 depletion in cells treated with CB5083 were limited to low glucose conditions, under which some nucleotide metabolites, alanine, and lactate accumulated in GCN2-depleted cells. Thus, GCN2 activity attenuated depletion of glycolytic metabolites under low glucose conditions but had a limited overall role in regulating intracellular metabolites.

### GCN2 is not required to attenuate protein synthesis under conditions of proteotoxic or nutrient stress

The translation initiation factor eIF2α is the only known direct GCN2 target, but there are several eIF2α kinases that are thought to respond to different stress situations [53]. Somewhat surprisingly, we observed that glucose depletion, which is predicted to result in protein misfolding and thus trigger eIF2α phosphorylation by PERK, substantially reduced the levels of eIF2α^Ser51^ phosphorylation at early (6 h) and late (36 h) time points in control cells. However, eIF2α^Ser51^ phosphorylation was largely maintained in GCN2-depleted cells at the 6 h time point (Fig. 5b). Glutamine depletion also resulted in lower levels of eIF2α phosphorylation, but the reduction in eIF2α^Ser51^ phosphorylation in control cells was less pronounced than in glucose-depleted cells at the early and late time point. Intriguingly, the level of eIF2α^Ser51^ phosphorylation was higher in GCN2-depleted cells than in control cells at the 36 h time point. In contrast to nutrient depletion, VCP/p97 inhibition in control cells grown in complete medium triggered the predicted [29, 30] increase in eIF2α^Ser51^ phosphorylation, particularly at the 36 h time point (Fig. 5b). However, this was not observed in GCN2-depleted cells. The effects of VCP/p97 inhibition on eIF2α^Ser51^ phosphorylation under glucose or glutamine-depleted conditions were very similar to the effects of nutrient depletion alone. Thus, eIF2α^Ser51^ phosphorylation that was triggered by VCP/p97 inhibition depended on GCN2, but only when glucose and glutamine were abundant. In contrast, unidentified mechanisms partly maintain eIF2α^Ser51^ phosphorylation early after glucose deprivation, and increase eIF2α^Ser51^ phosphorylation late after glutamine withdrawal, in GCN2-depleted cells.

We then studied the effects of VCP inhibition and GCN2 depletion on protein synthesis by immunoblotting for puromycinylated proteins (Fig. 5c). We found that glucose or glutamine depletion alone distinctly attenuated protein synthesis at the 36 h time point. VCP/p97 inhibition had a similar effect on protein synthesis, which was not enhanced by nutrient depletion. GCN2 depletion did not have a distinct quantifiable effect on protein synthesis under any of the stress conditions tested, although glucose depletion may have had a slightly more pronounced effect on protein synthesis in GCN2-depleted cells at the 36 h time point. Thus, despite different effects of nutrient limitations and VCP/p97 inhibition on eIF2α^Ser51^ phosphorylation, these metabolic stresses had a comparable effect on overall protein synthesis.

## Discussion

VCP/p97 regulates multiple cellular functions by virtue of its segregase activity [73]. Here, we provide evidence that VCP/p97 also acts as a central regulator of cellular metabolism, first by linking VCP/p97 with multiple metabolic processes on a transcriptional level, both across a large number of cancer cell lines and in primary tumour cells from a sizeable cohort of patients with MM. We then went on to uncover bidirectional functional interactions between VCP/p97 and cellular metabolism. We observed that glutamine depletion resulted in VCP/p97 upregulation both at the mRNA and protein level, and that VCP/p97 inhibition was linked to increased glutamine levels. The latter may be explained at least partly by the recent finding that VCP/p97 promotes degradation of GS [37]. A mechanistic link for the former is less apparent. However, given the central role of VCP/p97 in the ubiquitin-proteasome system (UPS), and considering that amino acid starvation increases not only cellular dependency on proteasomal protein degradation but also the synthesis of proteasome subunits [74, 75], it is not unlikely that induction of VCP/p97 is part of a ‘UPS response’ to starvation. In conjunction with the findings by Van Nguyen et al. [37] that VCP/p97 link glutamine metabolism with the immunomodulatory drug target CRBN, our data further highlight VCP/p97 as a promising anticancer drug target that may be particularly important in ‘glutamine-addicted’ [76] cancers.

We found that CB5083 treatment triggered significant increases in the levels of not just glutamine but most amino acids. Considering that proteasome inhibition decreases intracellular amino acids [30, 75], our findings raise the question why inhibition of VCP/p97 has the opposite effect. One possible explanation could be that VCP/p97 inhibition has a profound suppressive effect on de novo protein synthesis, and thus amino acid usage. This effect could also result in a general decrease in the demand for carbon and thus explain the accumulation of various metabolites that we observed. However, considering the wide range of cellular functions that are governed by VCP/p97, which include amino acid-regulating processes as specific as GS-mediated synthesis of glutamine and as general as autophagy, it seems likely that a complex array of effects on protein synthesis and degradation as well as metabolism are at play. While it is beyond the scope of this study to elucidate these processes, the data presented here underpin the biological importance of VCP/p97 in metabolism and provide the framework for further studies.

In this study, we also provide evidence for a cytoprotective role of GCN2. While GCN2 depletion did not have a detectable impact on cell viability or stress signalling in unstressed cells, we observed an incremental increase in the cellular requirement for GCN2 when cells were challenged with nutrient depletion, VCP/p97 inhibition, or both together. Our data are therefore compatible with observations that GCN2 is dispensable in mice unless they receive a low amino acid diet or are treated with l-asparaginase [77–80]. Together with reports that GCN2 promotes cancer cell survival and tumour angiogenesis under conditions of nutrient deprivation [61, 62], our observations provide further support for the notion that inhibition of GCN2 could be of therapeutic benefit. Moreover, GCN2 inhibition might be most effective in sensitising cancer cells in nutrient-poor tumour areas to drugs that affect amino acid metabolism, such as proteasome inhibitors or l-asparaginase, without relevant toxic effects on normal tissues.

While our observations on the cytoprotective role of GCN2 under different stress conditions are robust, it remains unclear precisely how GCN2 protects cells. To our surprise, we found that GCN2 depletion had limited effects on metabolites, including amino acids. The main effects we observed suggest that GCN2 activity attenuates depletion of glycolytic metabolites, which may support ongoing biosynthetic processes. We also found that GCN2 reduced the cellular requirement for survival-promoting ERK signalling and autophagy in glutamine-depleted and CB5083-treated cells. Our observations differ from previous reports that autophagy is activated downstream of GCN2, and that ERK mediates metabolic stress-induced cell death upstream of GCN2 [57–59, 63, 71]. While these discrepancies may be partly explained by differences in experimental conditions and cell types used, they also highlight the complexities of adaptive measure that may be triggered in cancer cells.

**Fig. 6:**
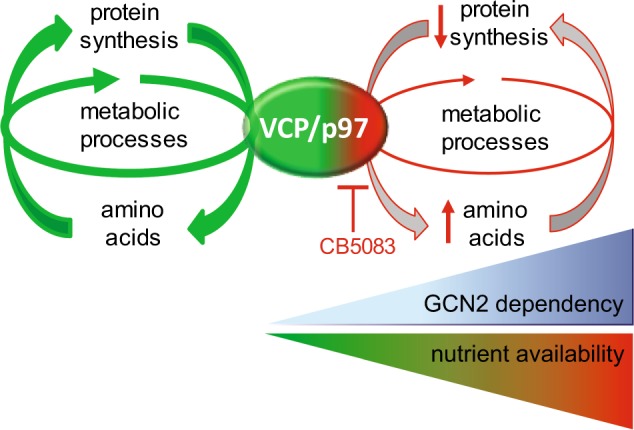
Proposed schematic model for the coordinated action of VCP/p97 and GCN2 to maintain metabolic and proteostatic homoeostasis. Under basal conditions, VCP/p97 promotes controlled protein turnover and metabolic homoeostasis. When VCP/p97 is functionally impaired, for example by pharmacological inhibition with CB5083, protein synthesis and metabolic processes are diminished, resulting in the accumulation of amino acids and metabolites. Additional nutrient shortages aggravate the proteostatic and metabolic imbalance and enhance cellular dependency on GCN2

In summary, we uncovered a role for VCP/p97 in cancer cell metabolism and in maintaining cancer cell ER protein homoeostasis under nutrient-restricted conditions. We also demonstrate that GCN2 signalling represents a mechanism by which cancer cells can cope with the combined action of VCP/p97 inhibition and nutrient deprivation (Fig. 6), by attenuating stress signalling and cell death, and by modulating ERK signalling, autophagy, and glycolytic metabolism. The findings are of relevance for anticancer therapies that target mechanisms of protein and metabolic homoeostasis. In particular, our observations highlight the need to investigate how the proteasome, a key partner of VCP/p97 in protein degradation, links protein and metabolic homoeostasis.

## Materials and methods

### Cells and cell culture reagents

A549 lung adenocarcinoma cells were a gift from Jane Mitchell (Imperial College London, London, UK). MCF-7 breast cancer cells were a gift from Dr. Ernesto Yagüe (Imperial College London, London, UK). PC3 and DU145 prostate cancer cells were a gift from Dave Carling (Imperial College London, London, UK). Saos-2 osteosarcoma cells and A172 glioblastoma cells were obtained from the European Collection of Authenticated Cell Cultures operated by Public Health England, UK. Cell culture identity was verified by short tandem repeat profiling provided by the University of Sheffield, UK, and the European Collection of Authenticated Cell Cultures operated by Public Health England, UK. A549 cells stably expressing inducible control shRNA or inducible shRNA targeting VCP/p97 (clones 4252–2 and 4252–3) were described before and were a kind gift from Dan Anderson (Cleave Biosciences) [29]. Cell lines were regularly tested for mycoplasma contamination using the MycoAlert Mycoplasma Detection Kit (Lonza, Cambridge, UK). Cells were maintained in Dulbecco’s modified Eagle’s medium (DMEM; Sigma-Aldrich, MO, USA) supplemented with 10% foetal bovine serum (FBS; Life Technologies, CA, USA) and 1% penicillin plus streptomycin solution (Sigma-Aldrich). For nutrient-depletion experiments, we used DMEM (Life Technologies, catalogue number A14430-01) supplemented with 2% phenol red solution (Sigma-Aldrich), 1% penicillin plus streptomycin, and 10% dialysed FBS (Gibco, Thermo Fisher, MA, USA, catalogue number A3382001). For ‘low glucose’ medium, we added 1 mM of d-(+) glucose solution (Sigma-Aldrich, catalogue number G844) and 2 mM l-glutamine (Sigma-Aldrich, catalogue number G7513). For ‘no glutamine’ medium, we added 25 mM of d-(+) glucose solution. For ‘complete’ medium, both 25 mM d-(+) glucose and 2 mM L-glutamine were added. All cells were cultured in a humidified atmosphere of 5% CO_2_ at 37 °C.

### Antibodies for immunoblotting and other reagents

Antibodies used for immuoblotting were as follows (catalogue numbers): eIF2α (9722S), β-tubulin (2146S), LC3B (2775S), PERK (5683S), p62 (5114S), ERK1/2 (137F5), phospho-ERK1/2 (Thr202/Tyr204) (9101), anti-rabbit IgG HRP-linked and anti-mouse IgG HRP-linked (7074S) (all from Cell Signalling Technology, MA, USA). Phospho-eIF2α (Ser51) (E90) (ab32157), VCP (ab11433) antibodies were from Abcam. PageRuler Plus Prestained Protein Ladder (Thermo Fisher) was used as a molecular weight marker. CB5083 (Stratech Scientific), Bafilomycin A1 (Sigma-Aldrich), Chloroquine (Sigma-Aldrich), AZD6244 (VWR), U0126 (Sigma-Aldrich), z-VAD-fmk (Promega), IPTG (VWR), and puromycin (InvivoGen) were dissolved in dimethylsulphoxide (DMSO) and stored at −20 °C.

### shRNA-mediated stable GCN2 knockdown

Stable shRNA-mediated GCN2 knockdown was achieved using pLKO.1 cloning vector (Addgene, Cambridge, MA, USA) with GFP replacing the puromycin insert. Briefly, pLKO stuffer was released upon digestion with EcoRI and AgeI (New England Biolabs, MA, USA). Oligos were annealed and ligated into the pLKO.1, producing a final plasmid expressing the shRNA of interest (shGCN2(1), shGCN2(2), and non-targeting shNTC (Supplementary Table 1)). DH5α cells (New England Biolabs) were transformed with ligation mix and plated on LB agar plates containing ampicillin. Positive clones were identified by sequencing with a pLKO.1 sequencing primer (5′ CAA GGC TGT TAG AGA GAT AAT TGG A 3′). Maxi prep using GeneJet Plasmid Maxiprep Kit (ThermoFisher) was carried out to produce large-scale quantities of the plasmid. Lentiviral particles were generated with HEK-293T (GenHunter) to carry out the viral transfection using X-treme Gene HP kit (Roche, Switzerland). Transfection efficiency was confirmed after 48 h; 72 h post-transfection virus was collected by ultracentrifugation. A549 cells were transduced and GFP-positive cells were sorted by FACS.

### Cell viability

Viable cells were quantified by flow cytometric analysis using Dead Cell Apoptosis Kit with Annexin V Alexa Fluor™ 488 and 7-amino-actimomycin D (7-AAD) according to the manufacturer instruction. Cells were analysed using a BD LSRFortessa™ (BD Biosciences). Cell viability was also assessed by RealTime-Glo™ MT Cell Viability Assay (Promega, Madison, WI, USA) according to the manufacturer’s instructions, using 3000 cells per well in 96-well white opaque-walled plates and a FLUOstar Omega microplate reader (BMG Labtech).

### Caspase activity assay

A total of 20,000 cells per well were seed in 96-well white opaque-walled plates. Following experimental treatment as indicated, Caspase-Glo® 3/7 Assay (Promega, catalogue number G8091) reagent was added according to the manufacturer’s instructions and luminescence was read on a Fluoroscan Ascent FL plate reader (Thermo Fisher Scientific).

### mRNA expression analysis by real-time RT-PCR

Cells were snap frozen using liquid nitrogen. RNA was extracted using the GeneJET RNA Purification Kit (Thermo Fisher) followed by removal of genomic DNA according to the manufacturer’s instructions. cDNA synthesis was performed using the RevertAid First Strand cDNA Synthesis Kit (Thermo Fisher) according to the manufacturer’s instructions using an Applied Biosystems 2720 Thermal Cycler (Life Technologies, Carlsbad, CA, USA). PCR reactions were performed on an Applied Biosystems StepOnePlus machine (Applied Biosystems, Foster City, CA, USA) using 10 μl SYBR Green JumpStart Taq ready Mix (Sigma-Aldrich), 0.3 μM sequence-specific primers (Supplementary Table 1), and 25 ng cDNA under standard conditions. Expression levels were quantified using the ΔΔ*C*_t_ or standard curve method with GAPDH as the control gene. GAPDH expression was confirmed to be stable in the different stress conditions tested, with minimal differences in *C*_t_ values that were not linked to a particular experimental condition.

### Western blotting

Whole-cell protein extracts were prepared on ice using a lysis buffer (Cell Signaling Technology) supplemented with Complete EDTA-free Protease Inhibitor Cocktail (Roche). After 10 min on ice and centrifugation at 4 °C, 14,000 rpm for 10 min, the supernatant was collected. The protein concentration was measured using a Bradford reagent (Bio-Rad Laboratories, CA, USA) according to the manufacturer's instruction. The proteins were denatured at 100 °C for 5 min with standard SDS-PAGE Loading Buffer and separated via 6–15% SDS polyacrylamide gel electrophoresis and transferred to a PVDF membrane, followed by blocking with TBS-T (0.1%) containing 5% non-fat milk for 1 h at room temperature, incubated with specific primary antibody at 4 °C for 16 h, and then incubated with secondary antibody labelled with horseradish peroxidase (HRP) for 1 h at RT. Finally, an electrochemiluminescence (ECL, GEHealthcare) system was used for detection of specific proteins. Intensity of the bands was measured using ImageJ software. The quantification reflects the relative amounts as a ratio of each protein band relative to the lane’s loading control.

### Analysis of protein synthesis by puromycin labelling

Semiquantitative monitoring of protein synthesis was carried out based on the previously described SUnSET method [81]. Briefly, newly synthesised peptides were labelled in cultured cells by the addition of puromycin (InvivoGen, San Diego, CA, USA); 5 μg/ml for 10 min before cells were collected and whole-cell extracts were prepared for immunoblotting as described above, using anti-puromycin antibody clone 12D10 (Merck Millipore, Darmstadt, Germany.

### Bioinformatic analyses

RMA-normalised expression data were downloaded from GEO (GSE36133, GSE15695) and the expression of VCP/p97 was correlated with all other probes. Multi-mapping probes were split, un-annotated probes were removed, and the most significant probe per gene was kept for downstream analysis. VCP/p97 gene correlation values were used as input to GSEA analysis against KEGG pathways using the R package ClusterProfiler [82]. Network graphs were produced with igraph using genes within the leading edge subset of the three most significantly enriched KEGG pathways [83].

### Statistical analysis

Unless stated otherwise, data on mRNA or protein expression and viability show means and standard error of the mean (SEM) from three independent experiments. Mann–Whitney *U* test to compare two sample means or one-way/two-way ANOVA with multiple comparison tests as indicated in the figure and table legends were performed using GraphPad Prism 7 software (GraphPad Software, CA, USA).

### GC-MS of intracellular metabolites

Intracellular metabolites were extracted from cultured cells by cold methanol quenching. Aqueous metabolites were separated from the intracellular extract using a 2:1:3 chloroform:methanol:water extraction method. The aqueous portion of the extract was separated and lyophilised in silanized 1.5 ml glass vials prior to analysis. Samples were derivatized for GC–MS using a two-step methoximation–silylation procedure. Samples were incubated with 20 μl of 20 mg/ml methoxyamine in anhydrous pyridine at 30 °C for 90 min, followed by the addition of 80 μl of *N*-methyl-*N*-trimethylsilyltrifluoroacetamide with 1% trimethylchlorosilane and incubating at 37 °C for 30 min. Samples were analysed on an Agilent 7890 gas chromatograph connected to an Agilent 5975 MSD using the FiehnLib settings [84]. GC–MS data were processed by deconvolution using AMDIS using the Fiehn library, followed by integration using GaVIN [85] based on the quantitation ion for each metabolite as taken from the Fiehn library. Background signals determined from an extraction blank were subtracted from the data, and were subjected to a smoothed-spline normalisation using repeat-injected pooled QC samples. Metabolite measurements were normalised to cell number. Statistical analyses of metabolome data were performed in R (3.3.0). The effects of treatment with the p97 inhibitor and with shGCN2 were evaluated through a two-way ANOVA for each of the three different media compositions separately. Effects of culture in low glucose or no glutamine media compared to full media were evaluated through a Student’s *t*-test. The calculated *p*-values were adjusted for multiple comparisons [86] to control the false discovery rate.

## Supplementary information

Supplementary Material
