# Supplementary material for: The coordinated action of VCP/p97 and GCN2 regulates cancer cell metabolism and proteostasis during nutrient limitation
Source: Oncogene. 2019 Jan 9;38(17):3216–31. doi: 10.1038/s41388-018-0651-z (PMC6756015; doi:10.1038/s41388-018-0651-z)
Supplement: Supplementary file 1 — Supplementary Material [file 41388_2018_651_MOESM1_ESM.pdf]

Supplementary Figure 1

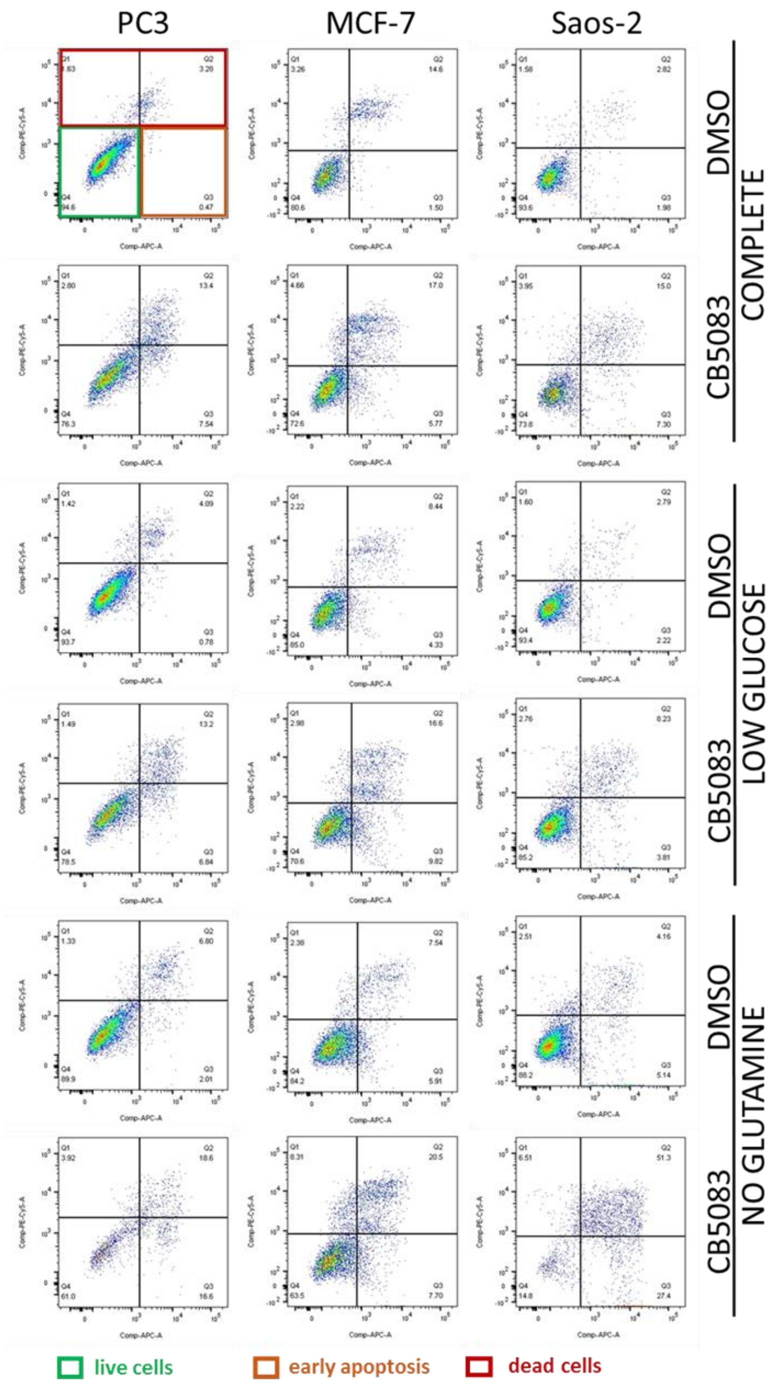

Glutamine depletion increases CB5083-induced cell death. Representative flow cytometry plots of Annexin V Alexa Fluor™ 488 and 7-amino-actimomycin D (7-AAD)-stained PC3, MCF-7 and Saos-2 cells grown in complete (25 mM glucose, 2 mM L-glutamine), low glucose (1 mM glucose, 2 mM L-glutamine) or no glutamine (25 mM glucose, 0 mM L-glutamine) medium and treated with vehicle (DMSO) or 1µM CB5083 for 48h. Lower left quadrant, live cells; lower right quadrant, early apoptotic cells; upper quadrants, late apoptotic/dead cells.

Supplementary Figure 2

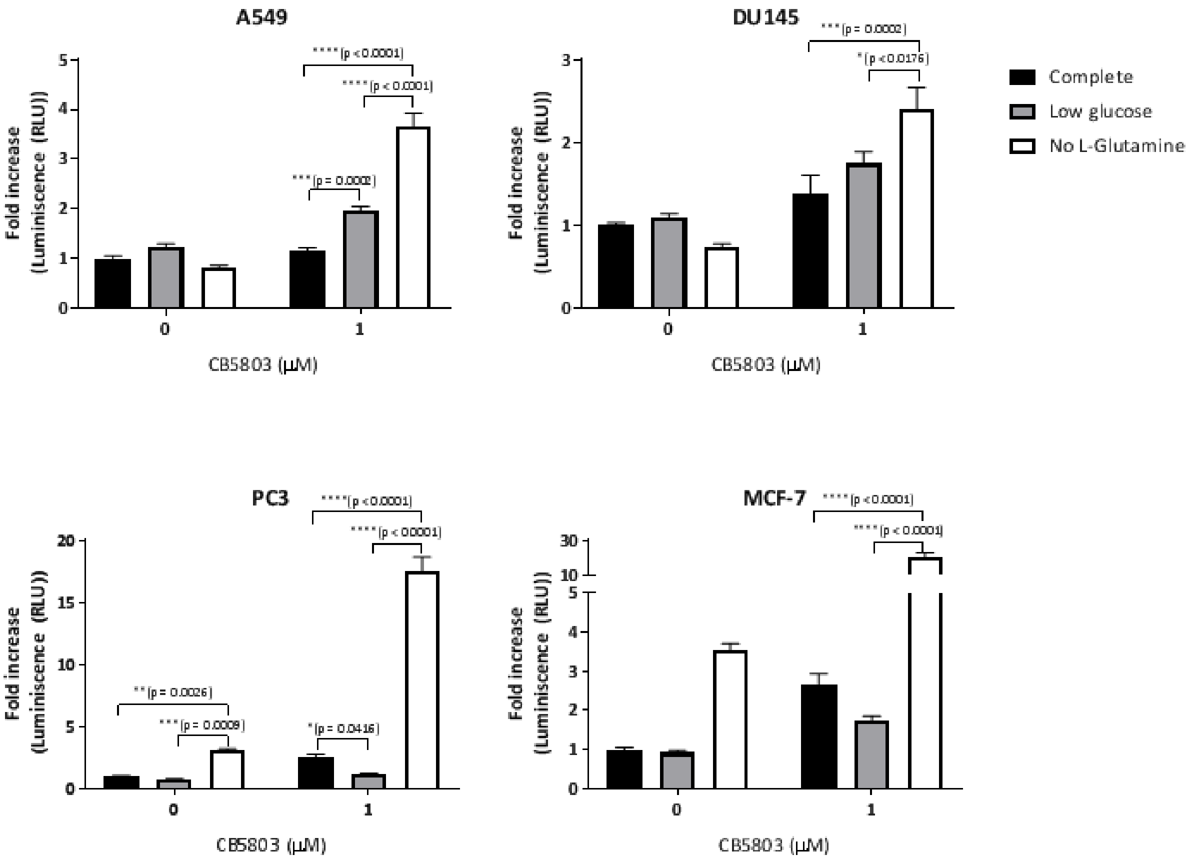

Nutrient depletion enhances CB5083-induced caspase activation. The indicated cells were treated with vehicle (DMS0, 0µM CB5083) or 1µM CB5083 in DMSO in complete (25 mM glucose, 2 mM L-glutamine), low glucose (1 mM glucose, 2 mM L-glutamine) or no glutamine (25 mM glucose, 0 mM L-glutamine) medium for 24h and caspase activation assessed using Caspase-Glo® 3/7 Assay (n=3, mean and SEM, two-way ANOVA and Tukey's multiple comparisons).

Supplementary Figure 3

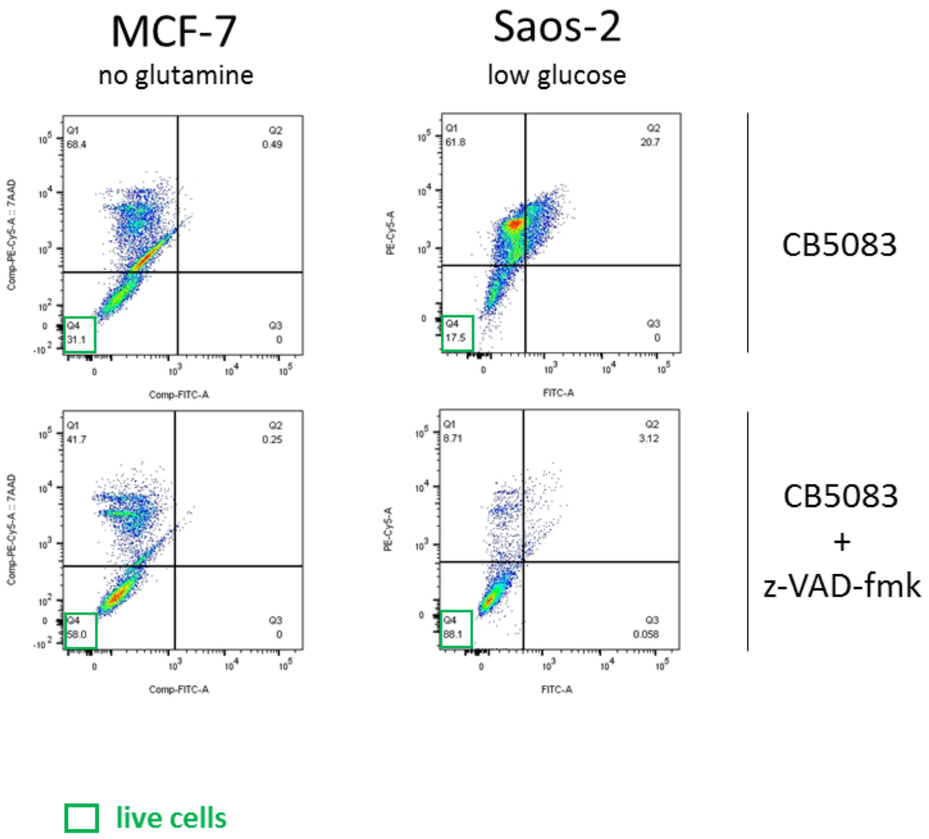

Cell death caused by nutrient depletion and CB5080-mediated VCP/p97 inhibition is partly caspase-dependent. MCF-7 and Saos-2 cells were treated with CB5083 (1µM) and the pan caspase inhibitor z-VAD-fmk (20µM) no glutamine or in low glucose (1mM) medium for 48h and viability assessed by flow cytometric analysis. Representative plots are shown to illustrate that cell death was partly (MCF-7, no glutamine+CB5083) or largely (Saos-2, low glucose+CB5083) caspase-dependent.

Supplementary Figure 4

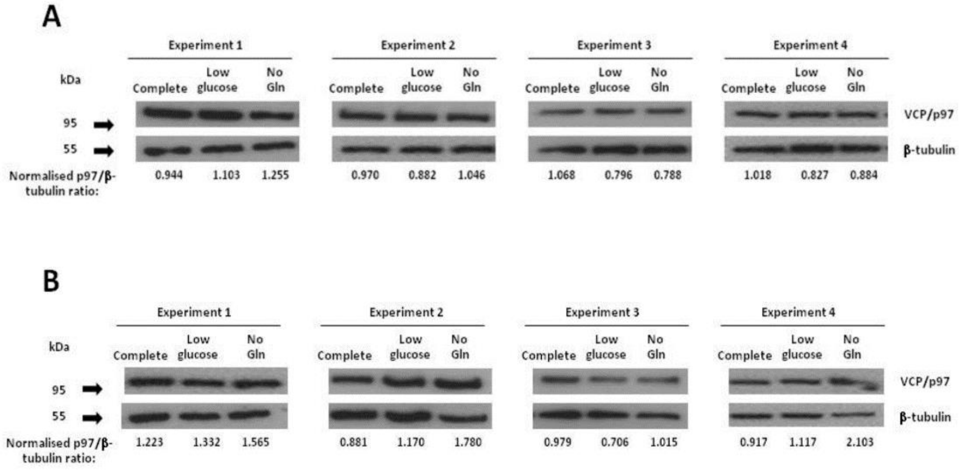

Glutamine depletion does not upregulate VCP/p97 protein expression. A549 cells were grown in complete (25 mM glucose, 2 mM L-glutamine), low glucose (1 mM glucose, 2 mM L-glutamine) or no glutamine (25 mM glucose, 0 mM L-glutamine) medium for either 8h (**A**) or 48h (**B**) in 4 independent experiments.

Supplementary Figure 5

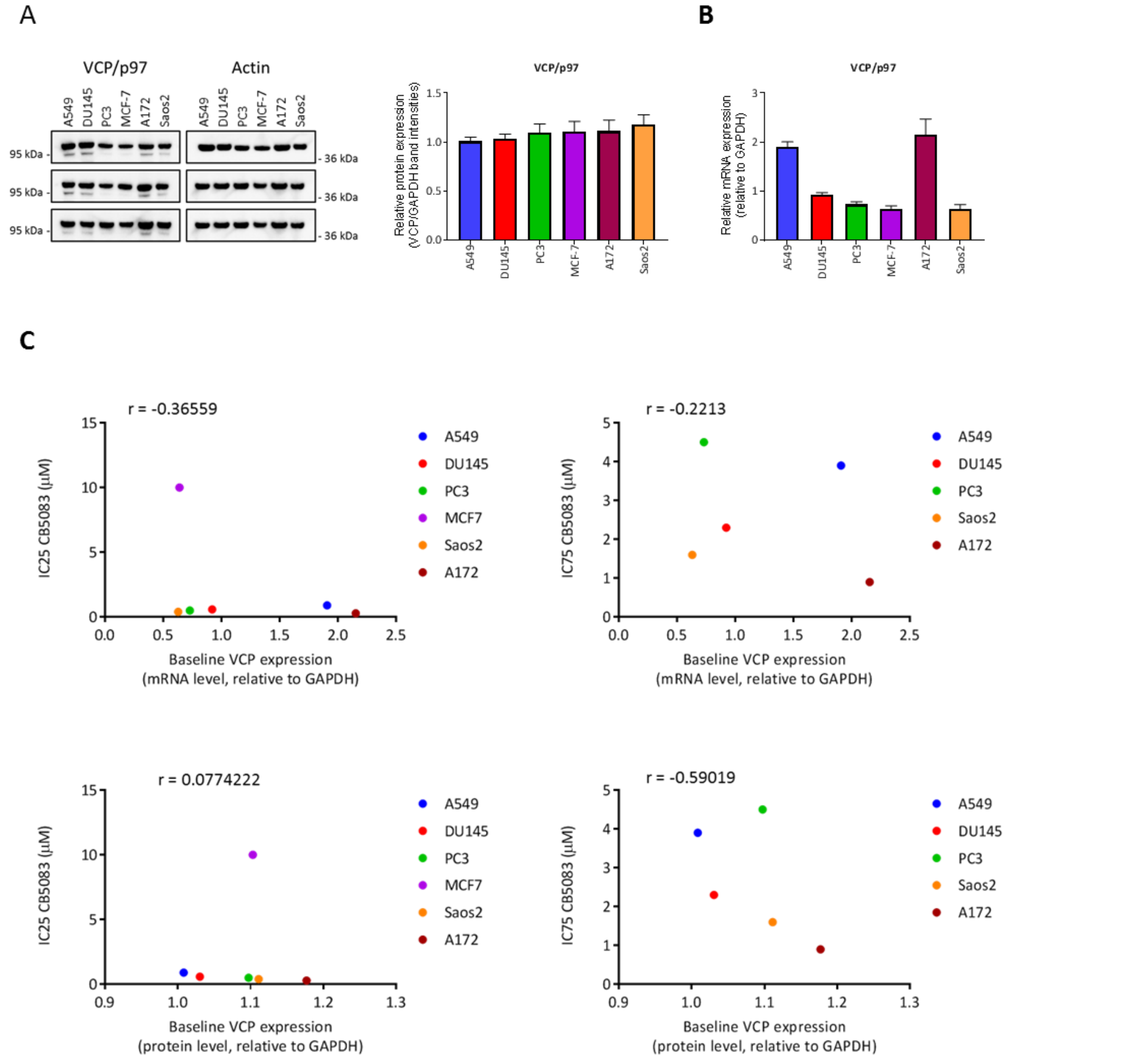

Baseline VCP/p97 protein and mRNA levels not correlate with sensitivity to CB5083. Protein **(A)** and mRNA **(B)** levels of VCP/p97 in a panel of cancer cells lines grown under basal (steady state) conditions in complete medium. The results of 3 independent experiments are shown (mean and SEM). Protein expression was quantified from immunblots using ImageJ. VCP/p97 band intensities were normalised against β-actin. **(C)** Correlation analysis of cell line viability following treatment with CB5083 and baseline protein or mRNA expression. IC25 and IC75 of the indicated cancer cell lines was assessed by RealTime-Glo™ MT Cell Viability Assay after treatment with CB5083 (0, 0.1, 0.3, 0.6, 1, 3.3, 6.6, and 10µM) for 48h. Cell death at 24h in response to 1 µM or 10µM CB5083 showed no correlation with baseline expression, either (r=-0.29 to 0.18; not shown).

Supplementary Figure 6

A

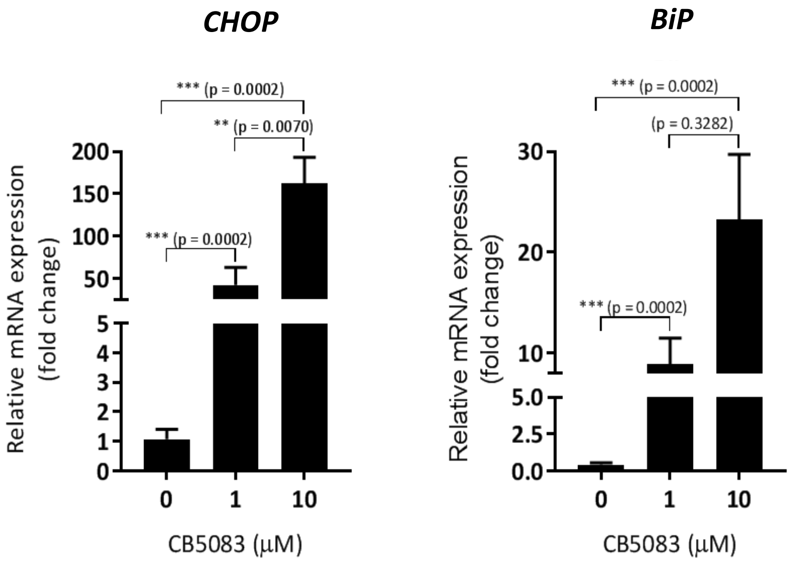

B

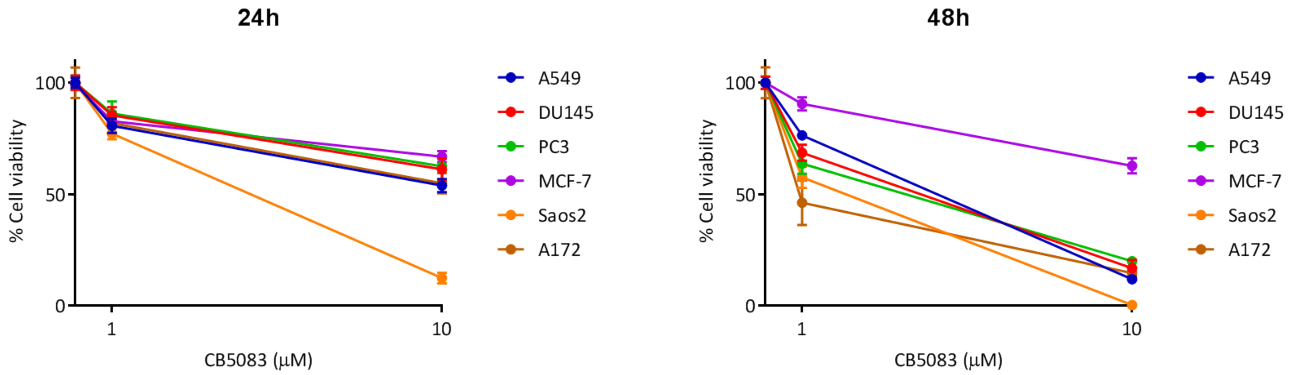

Concentration-dependent CB5083-induced proteotoxic stress gene expression and cell death. (A) A549 cells were grown in complete (25 mM glucose, 2 mM L-glutamine) medium and treated with CB5083 at the indicated concentrations for 16h (n=3, mean and SEM, Mann Whitney test). (B) Viability of the indicated cells was determined by RealTime-Glo™ MT Cell Viability Assay after treatment with CB5083 (1 or 10μM) for 24h or 48h (mean and SEM, n=3).

Supplementary Figure 7

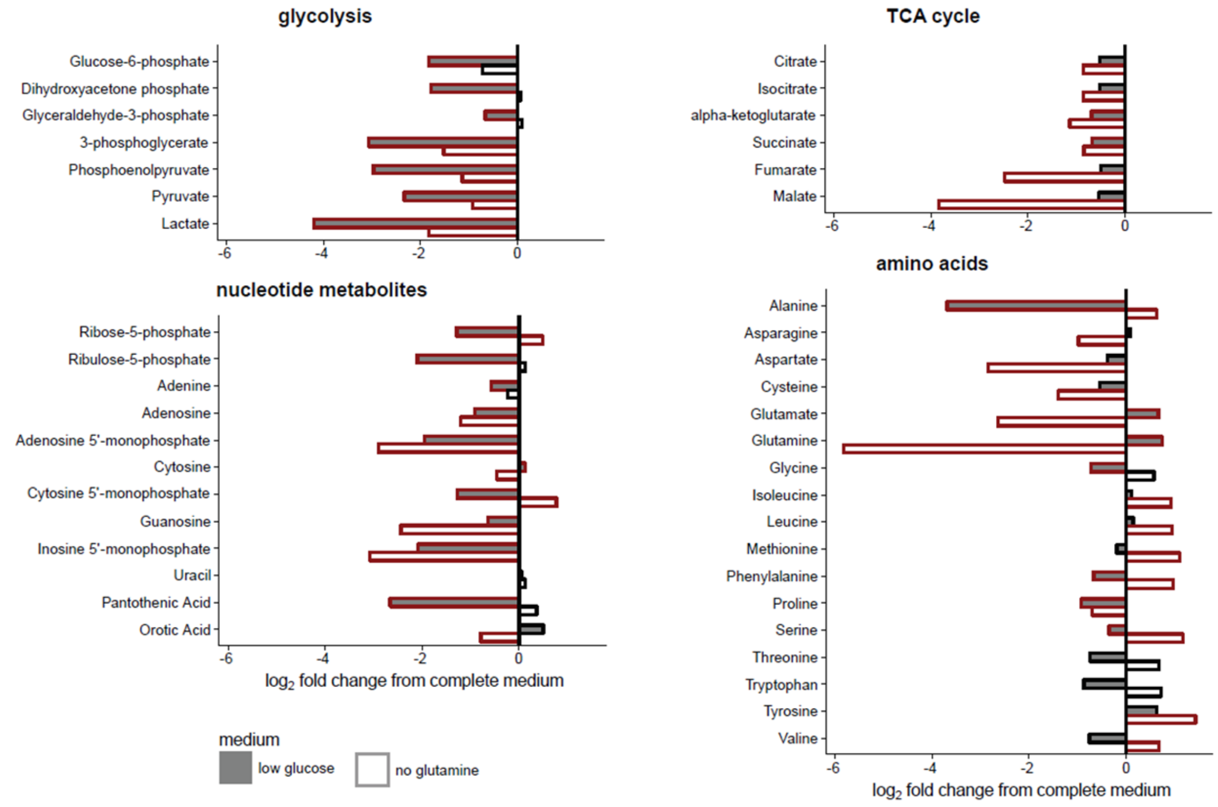

Metabolite responses to nutrient deprivation. A549 cells were grown in complete (25mM glucose, 2mM glutamine), low glucose (1mM glucose, 2mM glutamine), or no glutamine (25mM glucose, 0mM glutamine) medium for 16h followed by intracellular metabolite extraction and gas chromatography-mass spectrometry. Bar graphs show the effects of nutrient deprivation (fold change to complete medium). Comparisons with significant effects (two-way ANOVA, n=4 per experimental condition) are outlined in red.

Supplementary Figure 8

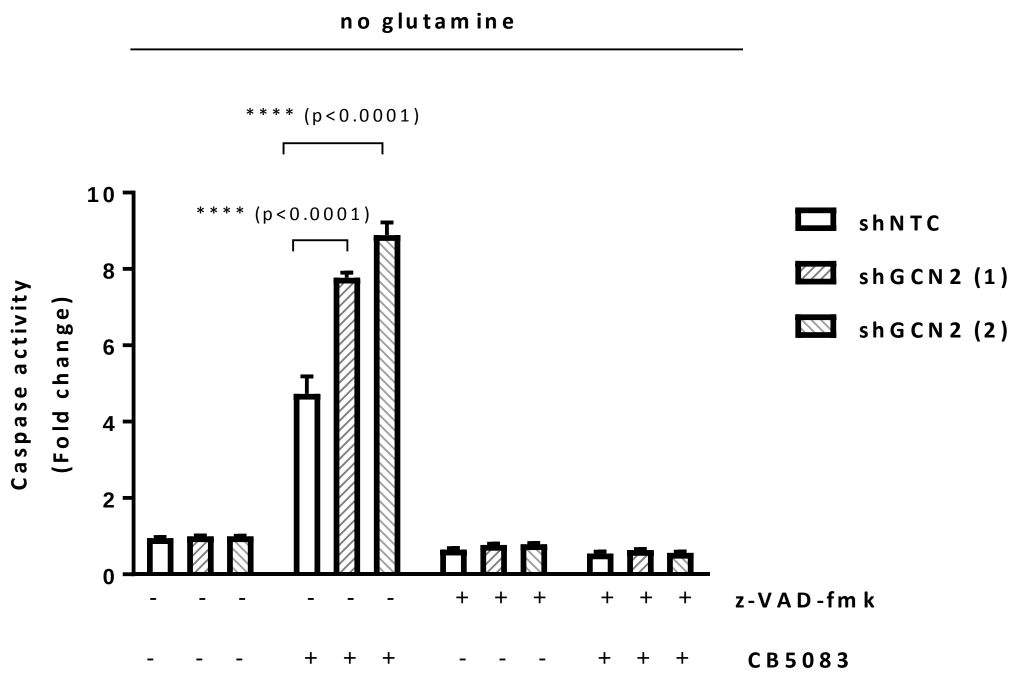

GCN2 depletion increases CB5083-induced caspase-activation. A549 cells stably expressing non-targeting shRNA (shNTC) or one of two shRNAs targeting GCN2 (shGCN2) grown in no glutamine medium (25mM glucose, 0mM glutamine) and treated with with z-VAD-fmk (20 µm) and CB5083 (1µM) as indicated for 24 hours followed by quantitation of caspase activity using the Caspase-Glo® 3/7 assay (n=3, mean and SEM, two-way ANOVA and Tukey's multiple comparisons test).

Supplementary Figure 9

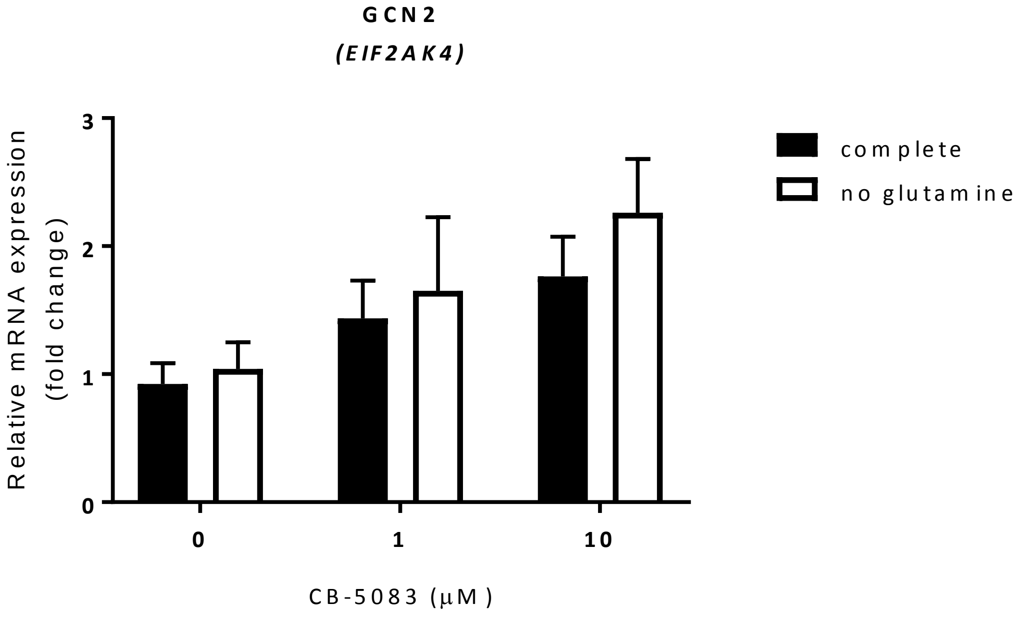

GCN2 (EIF2AK4) mRNA expression in response to VCP/p97 inhibition. A549 cells growing in complete (25mM glucose, 2mM glutamine) or no glutamine (25mM glucose, 0mM glutamine) medium were treated with vehicle (DMSO; 0 µM CB5083) or the indicated concentrations of CB5083 for 24h (n=3, mean and SEM, two-way ANOVA with Tukey's multiple comparisons [all p values >.05])

Supplementary Figure 10a

	Mean difference	95% CI difference	Significance	P value
**Experimental conditions (shNTC *vs* shGCN2)**				
control	-9.667	-20.45 to 1.119	ns	0.0918
control + AZD6244	-10.67	-21.45 to 0.1189	ns	0.0533
control + U0126	-10.67	-21.45 to 0.1189	ns	0.0533
control + CB5083	-23.67	-34.45 to -12.88	****	<0.0001
control + CB5083 + AZD6244	-19.33	-30.12 to -8.548	***	0.0006
control + CB5083 + U0126	-19	-29.79 to -8.214	***	0.0008

Statistical analysis of the effect of GCN2 depletion on cell viability. A549 cells stably expressing non-targeting shRNA (shNTC) or shRNA targeting GCN2 (shGCN2) grown under glutamine-depleted (0mM) conditions were treated with AZD6244 (4 µm), U0126 (10 µM) or CB5083 (1µM) for 24 hours followed by flow cytometric quantification of apoptotic cell death. Data shown are from 3 independent experiments (two-way ANOVA and Bonferroni multiple comparisons test. CI, confidence interval; ns, not significant; asterisks denote significance levels (***, <0.001; ****, <0.0001).

Supplementary Figure 10b

	Mean difference	95% CI difference	Significance	P value
**Experimental conditions (shNTC)**				
control ***vs*** AZD6244	-1.667	-10.35 to 7.013	ns	0.9895
control ***vs*** U0126	-6	-14.68 to 2.68	ns	0.2927
CB5083 ***vs*** CB5083 + AZD6244	-8	-16.68 to 0. 6801	ns	0.0818
CB5083 ***vs*** CB5083 + U0126	-4.667	-13.35 to 4.013	ns	0.5533
**Experimental conditions (shGCN2)**				
control ***vs*** AZD6244	-2.667	-11.35 to 6.013	ns	0.9235
control ***vs*** U0126	-7	-15.68 to 1.68	ns	0.1609
CB5083 ***vs*** CB5083 + AZD6244	-3.667	-12.35 to 5.013	ns	0.7668
CB5083 ***vs*** CB5083 + U0126	0	-8.68 to 8.68	ns	>0.9999

Statistical analysis of the effect of ERK inhibition on cell viability. A549 cells stably expressing non-targeting shRNA (shNTC) or shRNA targeting GCN2 (shGCN2) grown under glutamine-depleted (0mM) conditions were treated with AZD6244 (4 µm), U0126 (10 µM) or CB5083 (1µM) for 24 hours followed by flow cytometric quantification of apoptotic cell death. Data shown are from the same three independent experiments as the data shown in part A of the supplementary dataset (two-way ANOVA and Tukey's multiple comparisons test. CI, confidence interval; ns, not significant).

Supplementary Table 1

qRT-PCR primers

**BiP**	**F** GCCGTCCTATGTCGCCTTC<br>**R** TGGCGTCAAAGACCGTGTTC
**CHOP**	**F** AGCAGAGGTCACAAGCACCT<br>**R** TTCATGCTTGGTGCAGATTC
**ATG5**	**F** TTTGCATCACCTCTGCTTC<br>**R** TAGGCCAAAGGTTTCAGCTT
**ATG7**	**F** GGCCTTTGAGGAATTTTTTGG<br>**R** ACGTCTCTAGCTCCCTGCATG

shRNA sequences

**GCN1 (1)**	**F** CCGGGCCTAACTGGTGAAGAAGTATCTCGAGATACTTCTTCACCAGTTAGGCTTTTTG<br>**R** AATTCAAAAAGCCTAACTGGTGAAGAAGTATCTCGAGATACTTCTTCACCAGTTAGGC
**GCN2 (2)**	**F** CCGGCCAAAGGTCTATCAAATGAAACTCGAGTTTCATTTGATAGACCTTTGGTTTTTG<br>**R** AATTCAAAAACCAAAGGTCTATCAAATGAAACTCGAGTTTCATTTGATAGACCTTTGG
**NTC**	**F** CCGGAATTCTCCGAACGTGTCACGTCTCGAGACGTGACACGTTCGGAGAATTTTTTTG<br>**R** AATTCAAAAAAATTCTCCGAACGTGTCACGTCTCGAGACGTGACACGTTCGGAGAATT

Supplementary Table 2

ID	Description	group	enrichmentScore	NES	pvalue	p.adjust	qvalues
hsa01100	Metabolic pathways	Metabolism	0.263034271	1.61	0.00147	0.008321	0.0041671
hsa04151	PI3K-Akt signaling pathway	Signal transduction	0.325818548	1.81	0.00169	0.008321	0.0041671
hsa05200	Pathways in cancer	Cancers	0.378720774	2.12	0.00169	0.008321	0.0041671
hsa05165	Human papillomavirus infection	Infectious diseases	0.355326708	1.96	0.0017	0.008321	0.0041671
hsa05142	Chagas disease (American trypanosomiasis)	Infectious diseases	0.413832238	1.94	0.00177	0.008321	0.0041671
hsa04810	Regulation of actin cytoskeleton	Cell motility	0.448986716	2.35	0.00177	0.008321	0.0041671
hsa04144	Endocytosis	Transport and catabolism	0.41715452	2.22	0.00178	0.008321	0.0041671
hsa04015	Rap1 signaling pathway	Signal transduction	0.350263804	1.83	0.00178	0.008321	0.0041671
hsa04919	Thyroid hormone signaling pathway	Endocrine system	0.397319889	1.91	0.00179	0.008321	0.0041671
hsa04071	Sphingolipid signaling pathway	Signal transduction	0.409866078	1.97	0.00179	0.008321	0.0041671
hsa04140	Autophagy - animal	Transport and catabolism	0.413601893	2.01	0.00179	0.008321	0.0041671
hsa05145	Toxoplasmosis	Infectious diseases	0.379404039	1.8	0.0018	0.008321	0.0041671
hsa04371	Apelin signaling pathway	Signal transduction	0.378426662	1.86	0.0018	0.008321	0.0041671
hsa05212	Pancreatic cancer	Cancers	0.447586713	1.99	0.0018	0.008321	0.0041671
hsa04510	Focal adhesion	Cellular community	0.466208145	2.42	0.0018	0.008321	0.0041671
hsa04926	Relaxin signaling pathway	Endocrine system	0.423226291	2.05	0.0018	0.008321	0.0041671
hsa04390	Hippo signaling pathway	Signal transduction	0.432336644	2.14	0.0018	0.008321	0.0041671
hsa04072	Phospholipase D signaling pathway	Signal transduction	0.342938743	1.68	0.00181	0.008321	0.0041671
hsa05161	Hepatitis B	Infectious diseases	0.352577865	1.73	0.00181	0.008321	0.0041671
hsa04120	Ubiquitin mediated proteolysis	Folding, sorting and degradation	0.368642007	1.8	0.00181	0.008321	0.0041671
hsa04145	Phagosome	Transport and catabolism	0.479887053	2.38	0.00181	0.008321	0.0041671
hsa04142	Lysosome	Transport and catabolism	0.564420044	2.71	0.00181	0.008321	0.0041671
hsa01522	Endocrine resistance	Drug resistance	0.405350551	1.87	0.00181	0.008321	0.0041671
hsa04668	TNF signaling pathway	Signal transduction	0.405416535	1.91	0.00181	0.008321	0.0041671
hsa04360	Axon guidance	Development	0.394224179	2	0.00181	0.008321	0.0041671
hsa05205	Proteoglycans in cancer	Cancers	0.474022105	2.46	0.00181	0.008321	0.0041671
hsa04141	Protein processing in endoplasmic reticulum	Folding, sorting and degradation	0.58209225	2.93	0.00181	0.008321	0.0041671
hsa04150	mTOR signaling pathway	Signal transduction	0.345550043	1.71	0.00181	0.008321	0.0041671
hsa04722	Neurotrophin signaling pathway	Nervous system	0.384031074	1.84	0.00181	0.008321	0.0041671
hsa05169	Epstein-Barr virus infection	Infectious diseases	0.364250531	1.89	0.00181	0.008321	0.0041671
hsa04213	Longevity regulating pathway - multiple species	Aging	0.442101783	1.89	0.00181	0.008321	0.0041671

hsa04530	Tight junction	Cellular community	0.38181562	1.93	0.00181	0.008321	0.0041671
hsa04721	Synaptic vesicle cycle	Nervous system	0.468186095	2	0.00181	0.008321	0.0041671
hsa04910	Insulin signaling pathway	Endocrine system	0.376970555	1.85	0.00181	0.008321	0.0041671
hsa04210	Apoptosis	Cell growth and death	0.377043673	1.85	0.00181	0.008321	0.0041671
hsa05418	Fluid shear stress and atherosclerosis	Cardiovascular diseases	0.418044349	2.05	0.00181	0.008321	0.0041671
hsa05225	Hepatocellular carcinoma	Cancers	0.348847921	1.75	0.00182	0.008321	0.0041671
hsa05146	Amoebiasis	Infectious diseases	0.430783704	2	0.00182	0.008321	0.0041671
hsa04962	Vasopressin-regulated water reabsorption	Excretory system	0.530644044	2.08	0.00182	0.008321	0.0041671
hsa05120	Epithelial cell signaling in Helicobacter pylori infection	Infectious diseases	0.49275202	2.13	0.00182	0.008321	0.0041671
hsa05211	Renal cell carcinoma	Cancers	0.51239962	2.21	0.00182	0.008321	0.0041671
hsa05131	Shigellosis	Infectious diseases	0.56687864	2.42	0.00182	0.008321	0.0041671
hsa03050	Proteasome	Folding, sorting and degradation	0.714896308	2.8	0.00182	0.008321	0.0041671
hsa00510	N-Glycan biosynthesis	Glycan biosynthesis and metabolism	0.54731875	2.2	0.00182	0.008321	0.0041671
hsa05130	Pathogenic Escherichia coli infection	Infectious diseases	0.600016603	2.47	0.00182	0.008321	0.0041671
hsa04933	AGE-RAGE signaling pathway in diabetic complications	Endocrine and metabolic diseases	0.432256207	2	0.00182	0.008321	0.0041671
hsa05110	Vibrio cholerae infection	Infectious diseases	0.590727319	2.39	0.00182	0.008321	0.0041671
hsa04961	Endocrine and other factor-regulated calcium reabsorption	Excretory system	0.474484888	1.89	0.00183	0.008321	0.0041671
hsa04915	Estrogen signaling pathway	Endocrine system	0.411047653	1.9	0.00183	0.008321	0.0041671
hsa04066	HIF-1 signaling pathway	Signal transduction	0.423157954	1.96	0.00183	0.008321	0.0041671
hsa04520	Adherens junction	Cellular community	0.446867815	1.95	0.00183	0.008321	0.0041671
hsa00520	Amino sugar and nucleotide sugar metabolism	Carbohydrate metabolism	0.511746276	2.05	0.00183	0.008321	0.0041671
hsa04657	IL-17 signaling pathway	Immune system	0.409101731	1.88	0.00183	0.008321	0.0041671
hsa05412	Arrhythmogenic right ventricular cardiomyopathy (ARVC)	Cardiovascular diseases	0.437018786	1.91	0.00184	0.008321	0.0041671
hsa05100	Bacterial invasion of epithelial cells	Infectious diseases	0.543529882	2.41	0.00184	0.008321	0.0041671
hsa05215	Prostate cancer	Cancers	0.404491853	1.87	0.00185	0.008321	0.0041671
hsa04012	ErbB signaling pathway	Signal transduction	0.425581337	1.93	0.00186	0.008321	0.0041671
hsa04340	Hedgehog signaling pathway	Signal transduction	0.496434312	1.97	0.00186	0.008321	0.0041671
hsa04512	ECM-receptor interaction	Signaling molecules and interaction	0.441223949	1.99	0.00186	0.008321	0.0041671
hsa04350	TGF-beta signaling pathway	Signal transduction	0.442139875	1.99	0.00186	0.008321	0.0041671
hsa05132	Salmonella infection	Infectious diseases	0.538513608	2.43	0.00186	0.008321	0.0041671
hsa04917	Prolactin signaling pathway	Endocrine system	0.439266213	1.9	0.00186	0.008321	0.0041671

hsa05222	Small cell lung cancer	Cancers	0.44308434	2.02	0.00187	0.008321	0.0041671
hsa00532	Glycosaminoglycan biosynthesis - chondroitin sulfate / dermatan sulfate	Glycan biosynthesis and metabolism	0.630511199	2.03	0.00187	0.008321	0.0041671
hsa05213	Endometrial cancer	Cancers	0.445331243	1.87	0.00187	0.008321	0.0041671
hsa04370	VEGF signaling pathway	Signal transduction	0.451412294	1.9	0.00187	0.008321	0.0041671
hsa04540	Gap junction	Cellular community	0.509117656	2.31	0.00188	0.008321	0.0041671
hsa05020	Prion diseases	Neurodegenerative diseases	0.556482734	2.05	0.00188	0.008321	0.0041671
hsa04666	Fc gamma R-mediated phagocytosis	Immune system	0.428085014	1.94	0.00189	0.008321	0.0041671
hsa05414	Dilated cardiomyopathy (DCM)	Cardiovascular diseases	0.434159431	1.97	0.00189	0.008321	0.0041671
hsa00531	Glycosaminoglycan degradation	Glycan biosynthesis and metabolism	0.626306128	1.99	0.00191	0.008321	0.0041671
hsa03440	Homologous recombination	Replication and repair	-0.478921424	-1.9	0.00211	0.009035	0.0045245
hsa00670	One carbon pool by folate	Metabolism of cofactors and vitamins	-0.609028887	-2	0.00215	0.009083	0.0045487
hsa03010	Ribosome	Translation	-0.473174724	-2.4	0.00221	0.009196	0.0046053
hsa05322	Systemic lupus erythematosus	Immune diseases	-0.314272767	-1.6	0.00223	0.009196	0.0046053
hsa04014	Ras signaling pathway	Signal transduction	0.326139231	1.72	0.0035	0.011212	0.0056145
hsa04010	MAPK signaling pathway	Signal transduction	0.321304045	1.72	0.0035	0.011212	0.0056145
hsa04620	Toll-like receptor signaling pathway	Immune system	0.373764043	1.75	0.00353	0.011212	0.0056145
hsa05160	Hepatitis C	Infectious diseases	0.331517904	1.61	0.00355	0.011212	0.0056145
hsa04024	cAMP signaling pathway	Signal transduction	0.301137009	1.56	0.00357	0.011212	0.0056145
hsa04114	Oocyte meiosis	Cell growth and death	0.340468072	1.63	0.00358	0.011212	0.0056145
hsa04670	Leukocyte transendothelial migration	Immune system	0.36408264	1.73	0.00358	0.011212	0.0056145
hsa05203	Viral carcinogenesis	Cancers	0.285984272	1.48	0.00359	0.011212	0.0056145
hsa04916	Melanogenesis	Endocrine system	0.380337317	1.77	0.0036	0.011212	0.0056145
hsa04152	AMPK signaling pathway	Signal transduction	0.340010967	1.63	0.00362	0.011212	0.0056145
hsa04270	Vascular smooth muscle contraction	Circulatory system	0.328532988	1.57	0.00362	0.011212	0.0056145
hsa05230	Central carbon metabolism in cancer	Cancers	0.405400143	1.74	0.00362	0.011212	0.0056145
hsa05220	Chronic myeloid leukemia	Cancers	0.404755232	1.81	0.00362	0.011212	0.0056145
hsa01521	EGFR tyrosine kinase inhibitor resistance	Drug resistance	0.432622866	1.94	0.00362	0.011212	0.0056145
hsa04062	Chemokine signaling pathway	Immune system	0.298172473	1.52	0.00363	0.011212	0.0056145
hsa04022	cGMP-PKG signaling pathway	Signal transduction	0.323857695	1.63	0.00364	0.011212	0.0056145
hsa05223	Non-small cell lung cancer	Cancers	0.374616218	1.61	0.00365	0.011212	0.0056145
hsa05221	Acute myeloid leukemia	Cancers	0.390023527	1.68	0.00365	0.011212	0.0056145

hsa00010	Glycolysis / Gluconeogenesis	Carbohydrate metabolism	0.42100506	1.81	0.00365	0.011212	0.0056145
hsa00330	Arginine and proline metabolism	Amino acid metabolism	0.495424872	2.01	0.00365	0.011212	0.0056145
hsa05167	Kaposi's sarcoma-associated herpesvirus infection	Infectious diseases	0.306707282	1.57	0.00366	0.011212	0.0056145
hsa05216	Thyroid cancer	Cancers	0.467175278	1.76	0.00367	0.011212	0.0056145
hsa05133	Pertussis	Infectious diseases	0.425311847	1.89	0.00368	0.011212	0.0056145
hsa04730	Long-term depression	Nervous system	0.425167974	1.8	0.0037	0.011212	0.0056145
hsa00600	Sphingolipid metabolism	Lipid metabolism	0.465529495	1.83	0.0037	0.011212	0.0056145
hsa05410	Hypertrophic cardiomyopathy (HCM)	Cardiovascular diseases	0.411282767	1.85	0.00372	0.011212	0.0056145
hsa04912	GnRH signaling pathway	Endocrine system	0.355974934	1.61	0.00375	0.011212	0.0056145
hsa04392	Hippo signaling pathway - multiple species	Signal transduction	0.51232185	1.8	0.00376	0.011212	0.0056145
hsa04211	Longevity regulating pathway	Aging	0.360775032	1.64	0.00377	0.011212	0.0056145
hsa00514	Other types of O-glycan biosynthesis	Glycan biosynthesis and metabolism	0.540121187	1.78	0.00384	0.011297	0.0056571
hsa05217	Basal cell carcinoma	Cancers	0.386940355	1.65	0.00543	0.01508	0.0075516
hsa05224	Breast cancer	Cancers	0.317479954	1.57	0.00545	0.01508	0.0075516
hsa04137	Mitophagy - animal	Transport and catabolism	0.385471073	1.65	0.00545	0.01508	0.0075516
hsa00561	Glycerolipid metabolism	Lipid metabolism	0.39897486	1.7	0.00545	0.01508	0.0075516
hsa01230	Biosynthesis of amino acids	Metabolism	0.377508627	1.65	0.00549	0.01508	0.0075516
hsa05214	Glioma	Cancers	0.380844298	1.66	0.00549	0.01508	0.0075516
hsa05218	Melanoma	Cancers	0.384871958	1.71	0.00551	0.01508	0.0075516
hsa05210	Colorectal cancer	Cancers	0.39528033	1.73	0.00551	0.01508	0.0075516
hsa04914	Progesterone-mediated oocyte maturation	Endocrine system	0.352134236	1.6	0.0056	0.015171	0.0075971
hsa04974	Protein digestion and absorption	Digestive system	0.344469335	1.56	0.00567	0.015238	0.0076306
hsa04310	Wnt signaling pathway	Signal transduction	0.321139965	1.57	0.00719	0.019069	0.009549
hsa04261	Adrenergic signaling in cardiomyocytes	Circulatory system	0.321001157	1.58	0.00722	0.019069	0.009549
hsa05206	MicroRNAs in cancer	Cancers	0.290008304	1.48	0.00891	0.023267	0.0116515
hsa05164	Influenza A	Infectious diseases	0.29191827	1.48	0.00896	0.023267	0.0116515
hsa05231	Choline metabolism in cancer	Cancers	0.344827676	1.6	0.00914	0.023429	0.0117323
hsa04218	Cellular senescence	Cell growth and death	0.298168466	1.49	0.00917	0.023429	0.0117323
hsa00534	Glycosaminoglycan biosynthesis - heparan sulfate / heparin	Glycan biosynthesis and metabolism	0.487760291	1.66	0.00936	0.023598	0.011817
hsa00062	Fatty acid elongation	Lipid metabolism	0.491146399	1.67	0.00945	0.023598	0.011817
hsa04136	Autophagy - other	Transport and catabolism	0.457358775	1.64	0.00947	0.023598	0.011817
hsa04611	Platelet activation	Immune system	0.31708865	1.53	0.0107	0.026439	0.0132395

hsa04550	Signaling pathways regulating pluripotency of stem cells	Cellular community	0.307522643	1.51	0.01087	0.026494	0.0132675
hsa04728	Dopaminergic synapse	Nervous system	0.31868585	1.54	0.01089	0.026494	0.0132675
hsa04978	Mineral absorption	Digestive system	0.39772079	1.6	0.01275	0.03078	0.0154138
hsa04216	Ferroptosis	Cell growth and death	0.431051108	1.64	0.01306	0.031283	0.0156652
hsa03018	RNA degradation	Folding, sorting and degradation	-0.314913929	-1.5	0.01333	0.031692	0.0158704
hsa05166	HTLV-I infection	Infectious diseases	0.257024088	1.38	0.01396	0.032932	0.0164914
hsa00591	Linoleic acid metabolism	Lipid metabolism	-0.450961981	-1.6	0.01695	0.039676	0.0198686
hsa05219	Bladder cancer	Cancers	0.406482001	1.56	0.01708	0.039677	0.0198689
hsa03060	Protein export	Folding, sorting and degradation	0.483524846	1.61	0.01721	0.039682	0.0198715
hsa04068	FoxO signaling pathway	Signal transduction	0.302241922	1.47	0.01818	0.041616	0.02084
hsa05323	Rheumatoid arthritis	Immune diseases	0.322269348	1.46	0.0189	0.04295	0.021508
hsa00650	Butanoate metabolism	Carbohydrate metabolism	-0.44137446	-1.5	0.01948	0.043938	0.0220026
hsa05340	Primary immunodeficiency	Immune diseases	-0.398731221	-1.6	0.01969	0.044097	0.0220821
hsa04920	Adipocytokine signaling pathway	Endocrine system	0.339502427	1.47	0.02372	0.052736	0.0264084
hsa05134	Legionellosis	Infectious diseases	0.369244841	1.52	0.02403	0.053037	0.026559
hsa04610	Complement and coagulation cascades	Immune system	0.320032959	1.43	0.02545	0.055783	0.0279344
hsa04931	Insulin resistance	Endocrine and metabolic diseases	0.305078626	1.43	0.02737	0.059086	0.0295883
hsa04924	Renin secretion	Endocrine system	0.340695738	1.46	0.02742	0.059086	0.0295883
hsa00480	Glutathione metabolism	Amino acid metabolism	0.369646945	1.5	0.02762	0.059086	0.0295883
hsa05416	Viral myocarditis	Cardiovascular diseases	0.352971491	1.49	0.02773	0.059086	0.0295883
hsa00052	Galactose metabolism	Carbohydrate metabolism	0.424280997	1.52	0.02841	0.060126	0.0301091
hsa01200	Carbon metabolism	Metabolism	0.29012359	1.38	0.03058	0.064271	0.0321848
hsa05310	Asthma	Immune diseases	-0.411278848	-1.5	0.03165	0.066071	0.0330861
hsa00051	Fructose and mannose metabolism	Carbohydrate metabolism	0.423659129	1.53	0.03232	0.067025	0.0335638
hsa04612	Antigen processing and presentation	Immune system	0.317610266	1.41	0.03297	0.067912	0.0340081
hsa04966	Collecting duct acid secretion	Excretory system	0.432524832	1.5	0.03396	0.069499	0.0348027
hsa00515	Mannose type O-glycan biosynthesis	Glycan biosynthesis and metabolism	0.472086988	1.55	0.03455	0.069775	0.0349411
hsa00900	Terpenoid backbone biosynthesis	Metabolism of terpenoids and polyketides	0.472778143	1.56	0.03455	0.069775	0.0349411
hsa04621	NOD-like receptor signaling pathway	Immune diseases	0.261714175	1.32	0.03617	0.072568	0.0363394
hsa00860	Porphyrin and chlorophyll metabolism	Metabolism of cofactors and vitamins	0.384413309	1.46	0.03731	0.074386	0.0372501
hsa00562	Inositol phosphate metabolism	Carbohydrate metabolism	0.318737467	1.4	0.03867	0.076604	0.0383609
hsa00130	Ubiquinone and other terpenoid-quinone biosynthesis	Metabolism of cofactors and vitamins	0.58925102	1.6	0.03906	0.076881	0.0384994

hsa04921	Oxytocin signaling pathway	Endocrine system	0.268897543	1.33	0.03986	0.077944	0.0390319
hsa04971	Gastric acid secretion	Digestive system	0.31715954	1.4	0.04182	0.081269	0.0406969
hsa03460	Fanconi anemia pathway	Replication and repair	-0.333208042	-1.4	0.04555	0.087975	0.0440547
hsa05010	Alzheimer's disease	Neurodegenerative diseases	0.252805044	1.28	0.0483	0.092701	0.0464216
hsa04650	Natural killer cell mediated cytotoxicity	Immune diseases	-0.259922584	-1.3	0.04911	0.093667	0.0469055

Correlation of VCP/p97 mRNA expression across 917 CCLE cancer cell lines with KEGG pathways. The table shows 162 KEGG pathways that were significantly ($p<0.05$) correlated with VCP/p97 expression, ranked by significance level. Groups of particular relevance to metabolism or cancer biology are highlighted in different colours (blue, metabolism; light red, signal transduction; red, cancers; light green, transport and catabolism; green, folding, sorting and degradation.

Supplementary Table 3

ID	Description	setSize	Enrichment Score	NES	pvalue	p.adjust	qvalues
hsa01100	Metabolic pathways	1182	0.543900558	2.197665	0.000999	0.008884	0.005867
hsa04144	Endocytosis	232	0.395908166	1.552404	0.001011	0.008884	0.005867
hsa04714	Thermogenesis	213	0.568001437	2.223159	0.001017	0.008884	0.005867
hsa05169	Epstein-Barr virus infection	186	0.441370523	1.713041	0.001028	0.008884	0.005867
hsa05010	Alzheimer's disease	161	0.582670709	2.228397	0.001035	0.008884	0.005867
hsa05016	Huntington's disease	180	0.603090376	2.328134	0.001036	0.008884	0.005867
hsa00230	Purine metabolism	164	0.56291872	2.154103	0.001038	0.008884	0.005867
hsa04141	Protein processing in endoplasmic reticulum	158	0.529421038	2.017758	0.001042	0.008884	0.005867
hsa04932	Non-alcoholic fatty liver disease (NAFLD)	141	0.495637167	1.871583	0.00105	0.008884	0.005867
hsa03013	RNA transport	144	0.483604047	1.826904	0.001053	0.008884	0.005867
hsa04723	Retrograde endocannabinoid signaling	138	0.464310575	1.74652	0.001059	0.008884	0.005867
hsa04120	Ubiquitin mediated proteolysis	131	0.48589023	1.820736	0.001066	0.008884	0.005867
hsa05012	Parkinson's disease	126	0.662697332	2.480644	0.001066	0.008884	0.005867
hsa03010	Ribosome	113	0.59807638	2.226844	0.00107	0.008884	0.005867
hsa01200	Carbon metabolism	112	0.672532683	2.502762	0.001071	0.008884	0.005867
hsa00190	Oxidative phosphorylation	120	0.686226352	2.562279	0.001072	0.008884	0.005867
hsa03040	Spliceosome	118	0.455169366	1.699329	0.001072	0.008884	0.005867
hsa04142	Lysosome	118	0.483720057	1.80592	0.001072	0.008884	0.005867
hsa00240	Pyrimidine metabolism	93	0.6535051	2.383694	0.001087	0.008884	0.005867
hsa04146	Peroxisome	81	0.592988956	2.127979	0.001105	0.008884	0.005867
hsa03018	RNA degradation	76	0.494043188	1.751002	0.001115	0.008884	0.005867
hsa03008	Ribosome biogenesis in eukaryotes	70	0.628176632	2.208209	0.001121	0.008884	0.005867
hsa01230	Biosynthesis of amino acids	67	0.577351525	2.018091	0.001131	0.008884	0.005867
hsa00010	Glycolysis / Gluconeogenesis	66	0.601419049	2.097152	0.001138	0.008884	0.005867
hsa00280	Valine, leucine and isoleucine degradation	48	0.673776716	2.217781	0.001193	0.008884	0.005867
hsa00510	N-Glycan biosynthesis	47	0.642096449	2.102135	0.001202	0.008884	0.005867
hsa00520	Amino sugar and nucleotide sugar metabolism	46	0.657491617	2.147528	0.001202	0.008884	0.005867
hsa01212	Fatty acid metabolism	45	0.659954532	2.155408	0.001206	0.008884	0.005867
hsa03420	Nucleotide excision repair	43	0.648961192	2.105076	0.001209	0.008884	0.005867
hsa00071	Fatty acid degradation	42	0.623927964	2.013946	0.00122	0.008884	0.005867
hsa03050	Proteasome	42	0.782870427	2.526988	0.00122	0.008884	0.005867
hsa00620	Pyruvate metabolism	39	0.67680061	2.155643	0.001238	0.008884	0.005867
hsa00970	Aminoacyl-tRNA biosynthesis	41	0.795927945	2.552374	0.001239	0.008884	0.005867
hsa00380	Tryptophan metabolism	40	0.57493979	1.832054	0.001247	0.008884	0.005867
hsa03030	DNA replication	36	0.694509748	2.183299	0.001252	0.008884	0.005867
hsa00051	Fructose and mannose metabolism	32	0.592822861	1.818699	0.001276	0.008884	0.005867
hsa00640	Propanoate metabolism	32	0.701926814	2.153415	0.001276	0.008884	0.005867
hsa03410	Base excision repair	32	0.595866564	1.828037	0.001276	0.008884	0.005867
hsa00020	Citrate cycle (TCA cycle)	29	0.817607705	2.465696	0.001312	0.008884	0.005867
hsa00030	Pentose phosphate pathway	29	0.683253362	2.060518	0.001312	0.008884	0.005867
hsa00630	Glyoxylate and dicarboxylate metabolism	28	0.699398459	2.097811	0.001312	0.008884	0.005867

hsa00860	Porphyrin and chlorophyll metabolism	29	0.6375188	1.922594	0.001312	0.008884	0.005867
hsa03020	RNA polymerase	29	0.737555707	2.22428	0.001312	0.008884	0.005867
hsa00563	Glycosylphosphatidylinositol (GPI)-anchor biosynthesis	24	0.686207493	2.012844	0.001337	0.008884	0.005867
hsa03060	Protein export	23	0.612675674	1.775988	0.001344	0.008884	0.005867
hsa00900	Terpenoid backbone biosynthesis	22	0.712369588	2.044828	0.00135	0.008884	0.005867
hsa03430	Mismatch repair	22	0.674783864	1.93694	0.00135	0.008884	0.005867
hsa00062	Fatty acid elongation	21	0.682283628	1.938563	0.001372	0.008884	0.005867
hsa00040	Pentose and glucuronate interconversions	19	0.685215777	1.917521	0.001401	0.008884	0.005867
hsa00604	Glycosphingolipid biosynthesis - ganglio series	14	0.750553917	1.947358	0.001443	0.008884	0.005867
hsa01210	2-Oxocarboxylic acid metabolism	17	0.750846462	2.031616	0.001443	0.008884	0.005867
hsa00920	Sulfur metabolism	10	0.741390346	1.763277	0.001493	0.009013	0.005952
hsa04110	Cell cycle	122	0.433997111	1.626773	0.002125	0.012592	0.008316
hsa00983	Drug metabolism - other enzymes	60	0.525934279	1.797719	0.002296	0.013352	0.008818
hsa00500	Starch and sucrose metabolism	31	0.566350206	1.723317	0.002584	0.014752	0.009742
hsa03440	Homologous recombination	41	0.547082026	1.754377	0.003717	0.020844	0.013766
hsa03450	Non-homologous end-joining	12	0.72334123	1.79115	0.004405	0.024268	0.016027
hsa04721	Synaptic vesicle cycle	62	0.493699455	1.702524	0.004571	0.024749	0.016344
hsa00052	Galactose metabolism	30	0.558657026	1.695066	0.005188	0.027611	0.018235
hsa00480	Glutathione metabolism	49	0.515286308	1.707037	0.00591	0.03093	0.020426
hsa00410	beta-Alanine metabolism	31	0.546383399	1.662561	0.00646	0.033253	0.02196
hsa04150	mTOR signaling pathway	146	0.395788948	1.499957	0.007315	0.037045	0.024465
hsa05321	Inflammatory bowel disease (IBD)	61	-0.466417385	-2.09724	0.007752	0.038637	0.025516
hsa05310	Asthma	26	-0.492352028	-1.77852	0.008065	0.039567	0.02613
hsa00100	Steroid biosynthesis	18	0.623340148	1.715169	0.008559	0.041191	0.027203
hsa00053	Ascorbate and aldarate metabolism	14	0.663443118	1.721344	0.008658	0.041191	0.027203
hsa05133	Pertussis	70	-0.343922927	-1.57848	0.009091	0.042605	0.028137
hsa00534	Glycosaminoglycan biosynthesis - heparan sulfate / heparin	24	0.571528055	1.676456	0.009358	0.043213	0.028538
hsa04610	Complement and coagulation cascades	76	-0.435962527	-2.08864	0.009524	0.04334	0.028622
hsa00270	Cysteine and methionine metabolism	44	0.501859323	1.63815	0.010778	0.048042	0.031727
hsa00250	Alanine, aspartate and glutamate metabolism	35	0.520496108	1.623124	0.011364	0.048042	0.031727
hsa04640	Hematopoietic cell lineage	91	-0.331095362	-1.61065	0.011494	0.048042	0.031727
hsa04657	IL-17 signaling pathway	91	-0.417403927	-2.03051	0.011494	0.048042	0.031727
hsa04340	Hedgehog signaling pathway	45	-0.373017399	-1.56046	0.011561	0.048042	0.031727
hsa04064	NF-kappa B signaling pathway	90	-0.36399684	-1.76874	0.011628	0.048042	0.031727
hsa05146	Amoebiasis	95	-0.391240767	-1.91561	0.011628	0.048042	0.031727

Correlation of VCP/p97 mRNA expression with KEGG pathways in purified bone marrow tumour cells of 261 patients with multiple myeloma from the Myeloma IX trial. The table shows 76 KEGG pathways that were significantly ($p<0.05$) correlated with VCP/p97 expression, ranked by significance level. Metabolic pathways and pathways linked to protein processing in the ER and protein degradation are highlighted in blue and light red, respectively.
